# Conversion of Ku80 K568 crotonylation to SUMOylation facilitates DNA non-homologous end joining and cancer radioresistance

**DOI:** 10.1038/s41392-025-02210-1

**Published:** 2025-04-21

**Authors:** Hongling Zhao, Shanshan Gao, Yang Han, Dafei Xie, Lihui Xuan, Xin Huang, Jinhua Luo, Qian Ran, Gang Li, Hejiang Guo, Weixiang Hu, Jin Jia, Xiaochang Liu, Yuhao Liu, Jinpeng Tan, Chenjun Bai, Yongqing Gu, Teng Ma, Zhongjun Li, Hua Guan, Ruixue Huang, Ping-Kun Zhou

**Affiliations:** 1 Department of Radiation Biology, Beijing Key Laboratory for Radiobiology, Beijing Institute of Radiation Medicine, Beijing, China; 2 Department of Occupational and Environmental Health, Xiangya School of Public Health, Central South University, Changsha, Hunan Province China; 3 Laboratory of Radiation Biology, Laboratory Medicine Center, Department of Blood Transfusion, The Second Affiliated Hospital, Army Military Medical University, Chongqing, China; 4 School of Public Health, Hengyang Medical College, University of South China, Hengyang, Hunan Province China; 5 Cancer Research Center, Beijing Chest Hospital/Beijing Tuberculosis and Thoracic Tumor Research Institute, Capital Medical University, Beijing, China

**Keywords:** Cell biology, Cancer therapy

## Abstract

Chemo-/radioresistance of malignant tumors hampers cancer control and increases patient mortality. Efficient repair of damaged DNA is critical for the maintenance of genomic integrity and fidelity of genetic information. In reverse, increased DNA repair capability in cancer cells contributes to chemo-/radioresistance of malignant tumors. DNA double-strand break (DSB) is the most serious DNA damage and is also the principal molecular basis of radiotherapy. Upon DNA damage, the Ku80 is recruited and forms a critical DNA-PK complex at the DSB sites with Ku70 and the catalytic subunit (DNA-PKcs) to initiate DNA repair. How DNA-PK is assembled and activated is not fully understood. Based on the identification of radiation-reduced Ku80 K568 crotonylation through quantitative global lysine crotonylome analysis, we reveal that Ku80 K568 is crotonylated by p300-CBP-associated factor (PCAF). Upon DNA damage, the K568cr is decrotonylated by HDAC8 (Histone deacetylase 8). Decrotonylation of K568cr empties this site for the subsequent SUMOylation of Ku80 by CBX4. The conversion of Ku80 from K568 crotonylation to SUMOylation facilitates the assembly of DNA-PK complex and autophosphorylation of DNA-PKcs S2056, consequently activating the DSB repair. Moreover, mutation disrupting the post-translational modification (PTM) of Ku80 K568 site sensitizes cancer cells to radiotherapy in tumor-bearing nude mice models. This study elucidates the conversion model between two different forms of PTMs in the regulation of DNA-PK complex assembly and DSB repair, highlighting this model’s potential in controlling chemo-/radioresistance of malignant tumors, as well as expands the atlas of therapeutic targets.

## Introduction

DNA double-strand break (DSB) is the most deleterious DNA lesion and, if not repaired or if repaired incorrectly, can result in gene mutations, cell death, and the development of diseases, including cancer. To maintain genome stability, eukaryotic cells employ two main DSB repair mechanisms: homologous recombination (HR) and nonhomologous end-joining (NHEJ). NHEJ can be of two types, classical and alternate (Alt-EJ).^1^ In fact, we have known that the NHEJ pathway attends DSB ends during the cell cycle process, HR utilizes homologous DNA serving as a template to take action in error-free repair in the process of the S/G2 cell cycle phases.^2–5^

The NHEJ pathway is the predominant DSB repair pathway^6^; once DSBs occur, the Ku70/Ku80 heterodimer promptly binds to the broken DNA ends and recruits DNA-PKcs (DNA-dependent protein kinase catalytic subunit) to form an active Ku-DNA-PKcs complex, which is commonly referred to as the DNA-PK holoenzyme complex. After DNA-PK holoenzyme complex formation, DNA-PKcs phosphorylates itself and other NHEJ factors.^2,7^ DNA-PKcs kinase activity has been reported to have potential in efficiently DSB damage repair. A PQR cluster surrounds phosphorylated DNA-PKcs (Ser2056) to initiate NHEJ. Subsequently, it releases from broken DNA ends to enable subsequent repair proteins to access the damage sites.^8,9^ Next, DNA-PK activates and recruits Artemis nuclease for DSB end processing. Finally, with the assistance of XRCC4 (X-ray repair cross-complementing protein 4) and XLF (XRCC4-like factor), DNA ligase IV catalyzes the ligation of broken DNA ends.^2,3,6,8^ Although DNA-PK has been recognized as a DSB end-binding protein and repair-initiating complex for three decades,^10^ the mechanism through which DNA-PK components and functional activation are interrelated upon DNA damage remains unclear.

Ku80 (also named XRCC5), a component of the Ku heterodimer (Ku70, also named XRCC6 and Ku80 subunits), is a single-stranded DNA-dependent ATP-dependent helicase that plays a key role in DNA NHEJ which is able to bind to double-stranded DNA ends with high affinity in a sequence-independent manner by recruiting DNA-PK to DNA.^2,4,11^ Mutations or functional abnormalities of Ku80 are associated with a variety of diseases, including tumors.^12–14^ The DNA damage response (DDR) network comprises many post-translational modifications (PTMs) to proteins, which are known to have potential for affecting important biological events including sensing, signaling, and DNA damage repair. A recent report demonstrates that lactylation of MRE11 (double-strand break repair protein MRE11) at K673 by the CBP acetyltransferase promotes its binding to DNA, facilitating DNA end resection and HR.^15^ MRE11 SUMOylation in coordination with ubiquitylation is dynamically controlled by PIAS1 (E3 SUMO-protein ligase) and SENP3 (sentrin-specific protease 3) to facilitate DNA end resection and maintain genome stability.^16^ Ku80 can be ubiquitinated by multiple E3 ligases, including the RING finger domain-containing proteins (RNFs) RNF8^17^ and RNF126.^18^ Conversely, the ubiquitin carboxyl-terminal hydrolase L3 (UCHL3) can directly deubiquitylate Ku80.^19^ Ku80 ubiquitination and deubiquitylation facilitate the removal or retention of Ku80 from DSBs to complete DNA repair. In addition, Ku80 undergoes SUMO2/3-mediated SUMOylation at K307, which is correlated with apoptosis in colorectal cancer (CRC) cells under oxaliplatin stress,^20^ but whether SUMOylation is directly involved in DNA repair remains unclear.

Protein lysine crotonylation (Kcr), a newly identified PTM occurring in both core histone and nonhistone proteins, has been found to involve in many biological activities and function as regulator to affect physiological and pathological conditions in different cancer cells.^21–23^ PTMs are reversible, and Kcr is regulated by crotonyltransferase and decrotonylase.^22–24^ Previous studies have demonstrated that Kcr is involved in DNA DSBs repair.^25^ For example, H3K9cr level transiently decrease after laser microirradiation-induced DNA damage, providing the first indirect evidence of crotonylation involved in DDR;^26^ CDYL(chromodomain Y-like)-regulated Kcr of RPA1 is essential for regulation of HR pathway during DNA repair.^27^ Typically, while H2A at lysine 119 is decrotonylated and ubiquitinated at the same site, replication stress-induced transcription-replication conflicts can be solved, and genome stability can be protected.^28^ However, it is unknown whether the crotonylation of other proteins is directly involved in DNA damage repair and how it is regulated during the DDR, especially in the inter-regulation among DNA-PK components and functional activation for DNA DSBs repair.

In this study, we used a quantitative proteomics approach to obtain a global view of crotonylome alterations in response to ionizing radiation (IR), and a group of crotonylated lysine sites were identified in multiple DNA repair proteins associated with DNA DSBs and including components of the NHEJ pathway. Ku80 K568 was verified to be crotonylated by PCAF and decrotonylated by HDAC8. Decrotonylation of K568cr, which may have potential to affect the interaction between Ku80 and DNA-PKcs, was induced in cells upon IR-induced DNA damage. Importantly, decrotonylation of Ku80 K568cr is a prerequisite for subsequent CBX4-mediated SUMOylation at the same site. Moreover, K568 SUMOylation is required for DNA-PKcs S2056 autophosphorylation and NHEJ activation. The K568R mutation in Ku80 disrupts Ku80 and DNA-PKcs interaction and leads to DNA DSB repair deficiency and tumor sensitization to chemo-/radiotherapy. Taken together, our findings reveal a novel mechanism through which DNA-PK complex assembly and NHEJ initiation are regulated via the dynamic switching of Ku80 from crotonylation to SUMOylation. We introduce a new mechanism model of dynamic PTMs conversion for controlling DNA DSBs repair and even other critical cellular processes.

## Results

### Quantitative crotonylome analysis reveals altered protein crotonylation profiles in response to radiation-induced DNA damage

**Fig. 1:**
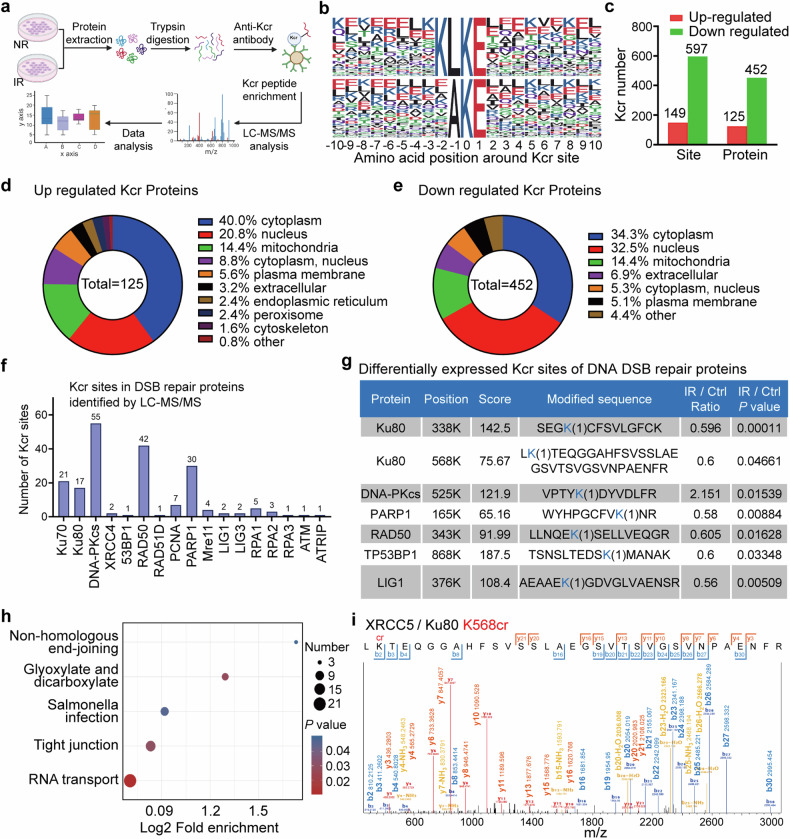
Identification of lysine crotonylation by quantitative crotonylome analysis in response to irradiation. **a** Flowchart of the experimental strategy for mapping the crotonylated peptides and sites using quantitative liquid chromatography (LC)‒tandem mass spectrometry (MS/MS) proteomic analysis. MCF-7 cells were irradiated with 8 Gy γ-rays (IR) or were nonirradiated (NR), and the cell lysates were harvested for crotonylome analysis after 4 h of culture. **b** Representative crotonylated peptide motif sequences and conservation of the amino acid sequences flanking the Kcr sites. **c** The number of differentially crotonylated lysine sites and proteins in irradiated cells compared to NR cells. **d** The subcellular distributions of upregulated crotonylated proteins. **e** The subcellular distributions of downregulated crotonylated proteins. **f** Total Kcr sites of proteins involved in DNA double-strand break (DSB) repair in irradiated and NR cells identified by LC-MS/MS. **g** The differentially expressed Kcr sites of DNA DSB repair proteins in irradiated cells. **h** Kyoto Encyclopedia of Genes and Genomes functional enrichment of the differentially expressed Kcr proteins after irradiation. **i** LC‒MS/MS spectrum of XRCC5/Ku80 peptides containing crotonylated K568

To determine how lysine crotonylation (Kcr) is globally involved in the regulation of the cellular response to IR-induced DNA damage, we used high-resolution liquid chromatography-tandem mass spectrometry (LC-MS/MS) to perform large-scale quantitative lysine crotonylome analysis in MCF-7 cells with or without γ-ray irradiation (Fig. 1a, b, Supplementary Fig. 1a). A total of 20910 Kcr sites across 3844 proteins were identified, among which a single Kcr site was identified for 979 proteins (25.47%), and more than six Kcr sites were identified for 1179 proteins (30.67%) (Supplementary Fig. 1b). The conservation of the amino acid sequences flanking the identified Kcr sites was evaluated against all human background sequences using IceLogo. Enrichment of negatively charged glutamate was found at the −2, +1, +2, +3, and +4 positions flanking the Kcr site (Supplementary Fig. 1c). Comparison with a recent report by Yu HJ et al. that there were 14,311 sites occurred lysine crotonylation within 3734 proteins in HeLa cells through large-scale analysis of protein crotonylation,^27^ of which 8086 Kcr sites were identified in both studies (Supplementary Fig. 1d). These 8086 Kcr sites account for 38.7% of the Kcr sites identified in this study and 56.5% of the Kcr sites identified in the report by Yu et al. indicating that the data are reliable.

Next, we identified proteins with altered Kcr levels in response to IR. We set the analysis threshold value of the cutoff ratio as >1.5 or <0.67 to represent the Kcr changes between non-irradiated (NR) and irradiated cells were significantly. Using this criteria, 149 Kcr sites in 125 proteins were found to be upregulated, whereas 597 Kcr sites in 452 proteins were downregulated in IR-induced cells (Fig. 1c, Supplementary Fig. 1e). The subcellular distribution of the differentially crotonylated proteins (Fig. 1d, e) shows that the majority of the upregulated Kcr proteins were distributed in the cytoplasm (40%), nucleus (20.8%) and mitochondria (14.4%), and the majority of the downregulated Kcr proteins were also distributed in the cytoplasm (34.29%), nucleus (32.52%) and mitochondria (12.17%).

To obtain an overview of the biological information of the differentially crotonylated proteins associated with the IR-induced DDR, Clusters of Orthologous Groups (COG)/Gene Ontology (GO) and Kyoto Encyclopedia of Genes and Genomes (KEGG) analyses were performed. COG functional analysis revealed that the most frequently identified class was [O] Posttranslational modification, protein turnover, and chaperones (54), followed by [U] Intracellular trafficking, secretion, and vesicular transport (53), [Z] Cytoskeleton (50), [K] transcription (48) and [J] Translation, ribosomal structure and biogenesis (48), and [A] RNA processing and modification(45) (Supplementary Fig. 1f). Nuclear genomic DNA is the primary target of IR,^3^ and DNA DSBs are the most critical type of DNA damage associated with cell death and the late stage effect of carcinogenesis.^4^ The COG functional classification also showed that 16 differentially expressed Kcr proteins were mapped to the [L] DNA replication, recombination, and repair category of information storage and processing (Supplementary Fig. 1f). In this study, we identified 191 crotonylated sites in 16 proteins that function in DNA DSB repair pathways (Fig. 1f, Supplementary Table 1), among which the crotonylation levels at seven sites in six proteins significantly changed after IR exposure (Fig. 1g). Most of these differentially expressed Kcr proteins are NHEJ pathway components of DSBs repair. Moreover, KEGG functional analysis also revealed that NHEJ was one of the five enriched functional pathways identified in the analysis of IR-induced differentially expressed Kcr proteins (Fig. 1h). The other four enriched pathways were involved in RNA transport, tight junctions, salmonella infection, and glyoxylate and dicarboxylate metabolism. DSB is a critical lethality DNA damage induced by IR.^2^ Ku80 is a key protein for recognizing DNA DSBs through forming heterodimer with Ku70 (also named XRCC6), and further binds DNA-PKcs/PRKDC to form the DNA-PK complex and initiate DSB repair through the NHEJ pathway. Our crotonylome profiling data demonstrated that K338 and K568 of Ku80 are potentially crotonylated, and the Kcr levels at both sites decreased after IR exposure (Fig. 1g, i, Supplementary Fig. 1g). Hence, it seems that crotonylation is an important form of PTMs for DNA repair proteins.

### Crotonylation at K568 of Ku80 is decreased in response to DNA damage

**Fig. 2:**
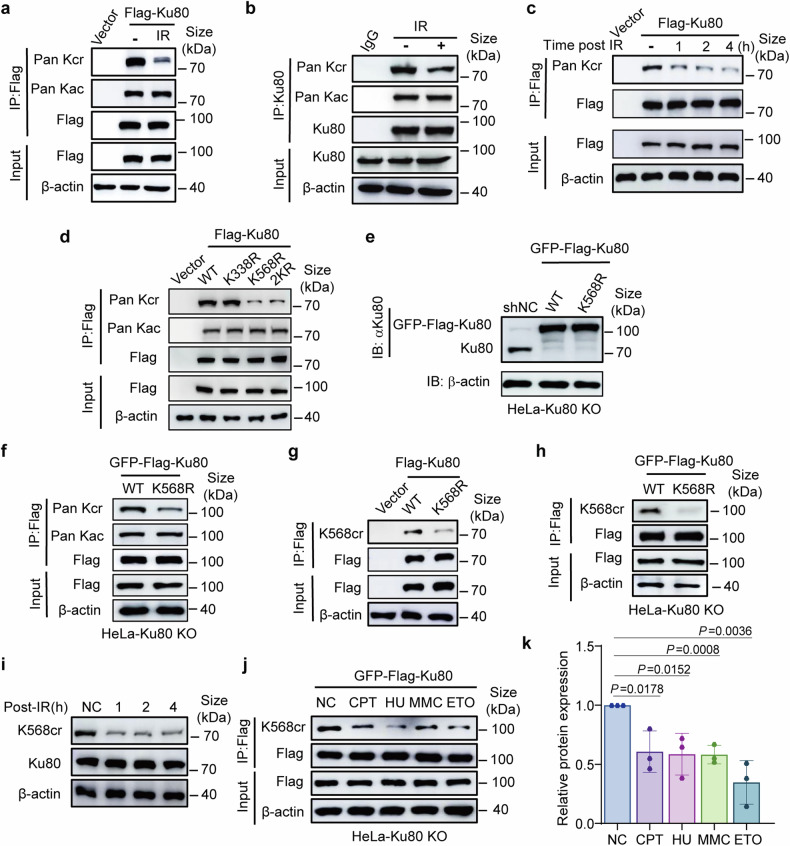
K568 crotonylation of Ku80 and its downregulation upon DNA damage. **a** An immunoprecipitation (IP) assay was performed to detect the crotonylation and acetylation levels of Ku80 in HEK-293T cells expressing the Flag-tagged Ku80 construct 4 h after 8 Gy γ radiation. **b** IP analysis of the crotonylation and acetylation levels of Ku80 in HeLa cells with or without 8 Gy of γ-ray irradiation. **c** IP analysis of the changes in Ku80 crotonylation 1 to 4 h after 8 Gy γ irradiation in HEK-293T cells expressing Flag-tagged Ku80 constructs. **d** The crotonylation levels of the Ku80 wild type (WT) and K338R and K568R mutants were determined by IP and western blotting with a pan-Kcr antibody in HEK-293T cells transfected with Flag-labeled Ku80 WT or mutant constructs. **e** Constructs of stable cell lines harboring exogenous GFP-Flag-wild-type Ku80 (WT) or GFP-Flag-mutant Ku80 (K568R) in Ku80-knockout HeLa cells. Western blot analysis was performed using the indicated antibodies. **f** The crotonylation of Ku80 was determined by IP with anti-Pan Kcr in stable cell lines of Ku80-knockout HeLa cells harboring exogenous GFP-Flag-WT or GFP-Flag-mutant Ku80 (K568R). **g**, **h** Verification of Ku80 K568cr using the specific anti-Ku80-K568cr antibody. IP was performed in HEK-293T cells transfected with Flag-labeled Ku80 WT or K568 mutant (**g**) and in stable cell lines of Ku80-knockout HeLa cells expressing exogenous GFP-Flag-Ku80 (WT) or mutant Ku80 (K568R) (**h**). **i** Western blot analysis using anti–Ku80–K568cr revealed time-dependent changes in Ku80 K568 crotonylation after 8 Gy γ radiation. **j** IP and WB analysis on the effects of different chemical DNA damaging agents on Ku80 K568 Kcr levels. Cells were treated with camptothecin (CPT, 1 μM), hydroxyurea (HU, 1 mM), mitomycin C (MMC, 5 μM), etoposide (ETO, 100 nM) or dimethyl sulfoxide solvent for control (NC) for 8 h, and western blotting was performed with the indicated antibodies on IP products of the Flag antibody. **k** The quantifications on the effects of different chemical DNA damaging agents on Ku80 K568 Kcr levels based on the analyses of WB gray values. Each data is the means and SD from three independent replications of WB experiments. A *p*-value less than 0.05 indicates a significant difference. β-actin was assayed to ensure equivalent loading and transfer

The quantitative mass spectrometry data suggested that K338 and K568 were the two key sites occurring crotonylation post radiation. Meanwhile Kcr levels of K338 and K568 were decreased in response to radiation (Fig. 1g). Given the critical role of Ku80 in initiating the NHEJ pathway of DNA DSB repair, we focused on this protein. The crotonylation status of Ku80 was confirmed by immunoprecipitation (IP) and western blotting with the antibody of crotonylated lysine (Pan Kcr). The results showed that the crotonylation levels of exogenously expressed Ku80 (Fig. 2a) and endogenously expressed Ku80 (Fig. 2b) dramatically decreased upon IR-induced DNA damage. However, IR exposure did not affect the total acetylation (Kac) level of Ku80 (Fig. 2a, b). A decrease in the Ku80 Kcr level occurred at least as early as 1 h post-irradiation (Fig. 2c, Supplementary Fig. 2a).

To verify the lysine site(s) at which crotonylation is decreased by irradiation, we constructed flag-tagged mutant vectors by replacing K338 or K568 with arginine (R) via site-specific mutagenesis. The WT and mutant vectors were transfected into HEK-293T cells. Western blotting analysis using the anti-Kcr pan-antibody indicated that the K568R point mutation affected the Ku80 crotonylation level (Fig. 2d). However, mutation of K338R seems barely affecting the total Ku80 Kcr level in cells treated with or without irradiation. Moreover, these point mutations had no effect on the Ku80 lysine acetylation (Kac) level (Fig. 2d). Therefore, we further focused our research on the K568 Kcr of Ku80. We next generated stable cell lines harboring exogenous GFP-Flag-tagged wild-type (WT) or K568R mutant (K568R) Ku80 in Ku80-knockout (KO) HeLa cells (HeLa-Ku80 KO) (Fig. 2e, Supplementary Fig. 2b). Kcr levels were largely decreased in the Ku80 KO HeLa cells expressing the exogenous Ku80 K568R mutant (Fig. 2f).

We further generated polyclonal antibodies specifically recognizing the crotonylated K568 residue of Ku80 (K568cr). Targeting the K568 site with or without Kcr modification and dot blotting assays using corresponding peptides, we verified its specificity (Supplementary Fig. 2c), and can be used in the following experiments. An IP plus western blotting assay using a K568cr-specific antibody verified that the crotonylation level of exogenous Ku80-K568R mutant was lower than that of Ku80-WT in HEK-293T cells (Fig. 2g) and in HeLa-Ku80 KO (Fig. 2h). Using this anti-K568cr specific antibody, we further confirmed the decrease of Ku80 Kcr in the lysates from the γ-ray-irradiated HeLa cells (Fig. 2i) as well as in the anti-Flag IP products from the irradiated cells expressing exogenous GFP-Flag-tagged Ku80 (Supplementary Fig. 2d). Moreover, we investigated the effect of various DNA-damaging chemical agents on the levels of Ku80 K568cr. As shown in Fig. 2j, k, the crotonylation level decreased in the cells after treatment with camptothecin (CPT), hydroxyurea (HU), mitomycin C (MMC), or etoposide (ETO). Together, these results demonstrate that the K568 site of Ku80 is crotonylated in cells under normal growth conditions and is downregulated upon the induction of DNA damage.

### HDAC8 and PCAF coregulate Ku80 crotonylation

**Fig. 3:**
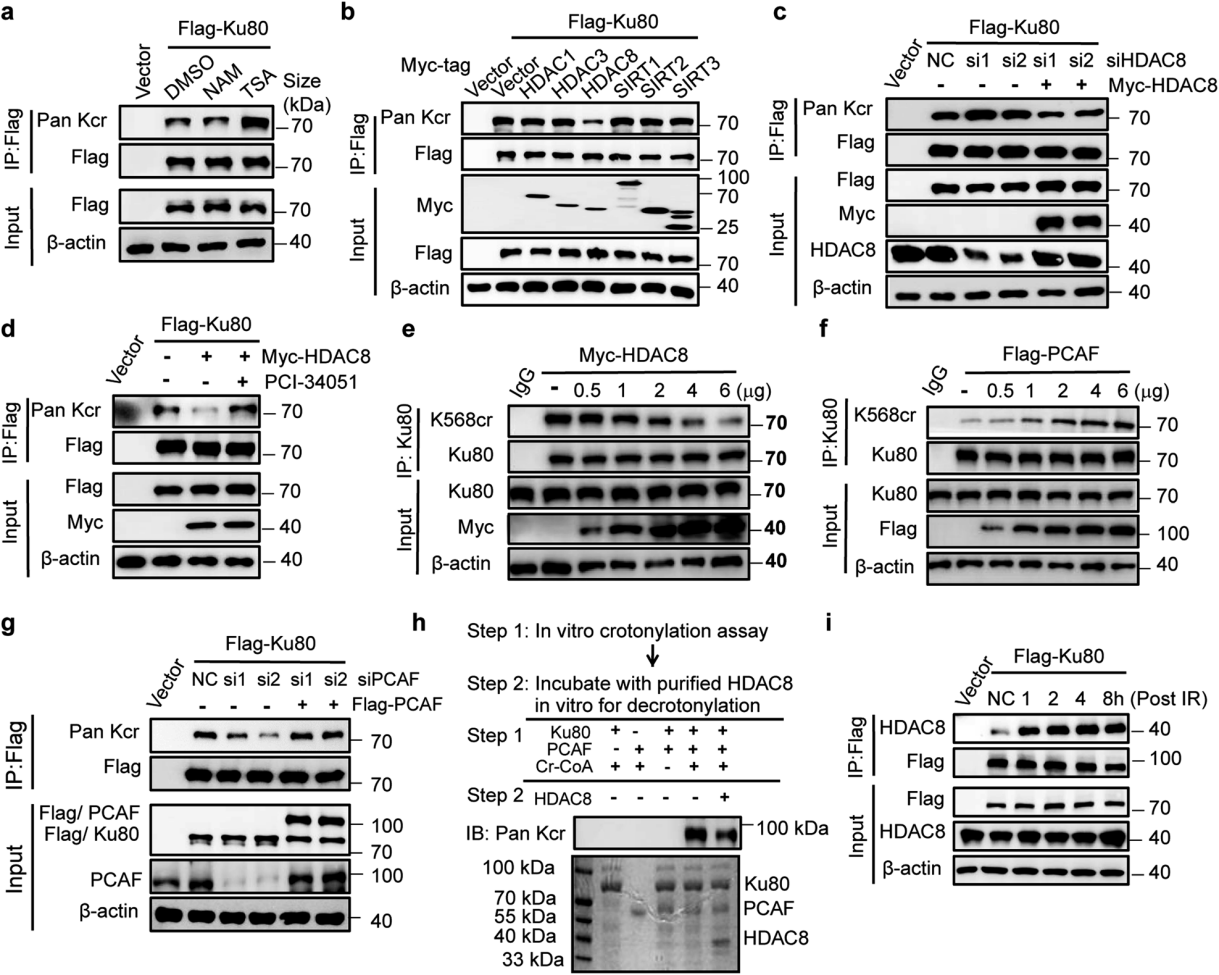
HDAC8 and PCAF coregulate the crotonylation of Ku80. **a** Functional analysis of the effects of TSA (1 μM, 10 h) and NAM (10 mM, 10 h) treatment on the decrotonylation of Ku80. **b** IP and western blotting were performed to identify the key regulating decrotonylase for Ku80 with the indicated antibodies. **c** HEK-293T cells expressing exogenous Flag-Ku80 were transfected with the indicated siRNAs and plasmids. Later, Ku80 crotonylation degrees were evaluated on IP products of the Flag antibody by immunoblotting. **d** Treatment with the HDAC8-specific inhibitor PCI-34051 (10 μM, 6 h) increased the crotonylation level of Ku80 in HEK-293T cells. **e** HDAC8 disrupted Ku80 crotonylation in a dose-dependent manner in HeLa cells. **f** PCAF increased Ku80 crotonylation in a dose-dependent manner in HeLa cells. **g** Impaired Ku80 crotonylation by PCAF siRNA is reversed by transfection of PCAF siRNA-resistant plasmids in HEK-293T cells. **h** PCAF crotonylates and HDAC8 decrotonylates Ku80 in vitro cell-free reaction assay. **i** Co-IP with DNase I treatment shows increased interaction between Ku80 and the decrotonylase HDAC8 after radiation exposure. Three independent replications of WB experiments. β-actin was assayed to ensure equivalent loading and transfer

It has been known that protein crotonylation and acetylation may share multiple acetylases and deacetylases.^29,30^ We next attempted to identify whether and which HDACs or SIRTs (NAD-dependent protein deacetylase sirtuin) acts as the decrotonylase for Ku80 decrotonylation and thus might be involved in the regulation of IR-induced Ku80 Kcr. When we treated the cells separately with the SIRT family inhibitor nicotinamide (NAM) or HDAC inhibitor trichostatin A (TSA), we found that only the HDAC inhibitor TSA increased the Ku80 crotonylation level (Fig. 3a). Moreover, when we transiently expressed different HDACs in combination with Flag-tagged Ku80 to determine the Ku80 crotonylation level, only HDAC8 largely abolished Ku80 crotonylation (Fig. 3b). However, this effect was reversed upon HDAC8 knockdown (Fig. 3c) or treatment with the HDAC8-specific inhibitor PCI-34051 (Fig. 3d). In addition, as HDAC8 expression increased continuously, Ku80 crotonylation levels gradually decreased (Fig. 3e). These results suggest that HDAC8 is a specific Ku80 decrotonylase.

Next, to identify the crotonylase for Ku80 Kcr, we first developed multiple overexpression of acetyltransferases in HEK-293T cells. Then, through IP assay, as well as western blotting analysis, the outcomes were strongly suggested that PCAF acts as the crotonylase of Ku80 Kcr (Supplementary Fig. 3a). The crotonylation level of endogenous Ku80 gradually increased as PCAF expression increased continuously (Fig. 3f). Ku80 crotonylation decreased in PCAF-knockdown cells, and this was reversed in cells transfected with the PCAF siRNA-resistant plasmids (Fig. 3g). These data illustrated PCAF is the Ku80 crotonylase. To confirm these results, we further used the purified Ku80 WT to test its crotonylation alteration in vitro. Figure 3h showed Ku80 can be crotonylated by PCAF, and subsequently decrotonylated by HDAC8. These results confirmed that HDAC8 acts as a decrotonylase for Ku80 and that PCAF acts as a crotonyltransferase for Ku80.

To further determine how radiation exposure results in decreased crotonylation of the Ku80 K568 site, we further examined the interaction between Ku80 and the crotonylation mediators PCAF and HDAC8 after radiation exposure. Co-IP assays revealed that irradiation does not change the general interaction of Ku80 and PCAF (Supplementary Fig 3b). Nevertheless, the interaction between Ku80 and HDAC8 dramatically increased after irradiation (Fig. 3i). Therefore, the decreased crotonylation of Ku80 could be attributed to the IR-induced interaction of Ku80 and HDAC8, which catalyzes Ku80 decrotonylation.

### The K568R mutation of Ku80 affects its interaction with DNA-PKcs and attenuates the autophosphorylation of DNA-PKcs S2056

To determine the biological significance of K568 crotonylation on Ku80 function in response to DNA damage, we performed a laser micro-irradiation experiment in stable cell lines harboring exogenous wild-type Ku80 (WT) or mutant Ku80 (K568R) in Ku80-knockout (KO) HeLa cells. The data showed that K568R mutation did not impair the recruitment of Ku80 to DNA damage sites induced by laser micro-irradiation (Supplementary Fig. 4a). Moreover, neither HDAC8 specific inhibitor PCI-34051 treatment nor PCAF knockdown affected the recruitment of Ku80 to DNA damage sites induced by laser micro-irradiation (Supplementary Fig. 4b, c), implying that the status of K568 PTM does not affect Ku80 recruitment to DNA damage. To corroborate the aforementioned findings, we further analyzed the recruitment of Ku80 to chromatin in cells. Upon IR-induced DNA damage, the amount of Ku80 binding to chromatin increased as expected, which was also not affected by the treatment of HDAC8 specific inhibitor PCI-34051 (Supplementary Fig. 4d) or PCAF knockdown mediated by siRNA (Supplementary Fig. 4e). These findings indicate that Ku80 crotonylation status does not affect the recruitment of Ku80 to DNA damage sites.

**Fig. 4:**
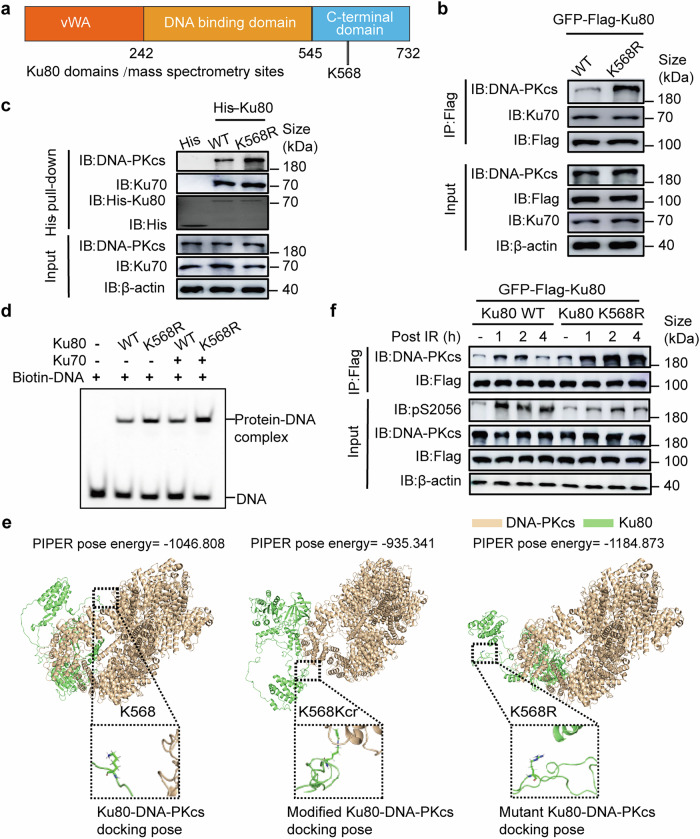
Permutation of Ku80 lysine 568 with arginine (K568R) affects the interaction of Ku80 with DNA-PKcs. **a** A schematic of Ku80 domains and crotonylation site. **b** Co-IP and western blotting were performed to identify the binding of Ku80 to DNA-PKcs and Ku70 with the indicated antibodies in stable cell lines of Ku80-knockout HeLa cells expressing exogenous Ku80 (WT) or permutated Ku80 (K568R). **c** His-pull-down assays were performed using His-Ku80 WT or K568R. Samples were separated by SDS‒PAGE. Samples were detected by Western blotting. **d** EMSAs were performed with the dsDNA, Ku80 WT, or K568R and Ku70 proteins. **e** The three-dimensional structure of crotonylated and permutated (R) Ku80 interacting with DNA-PKcs compared to that of the original Ku80 at the K568 site. **f** Co-IP and western blot assays were performed in stable cell lines bearing exogenous wild-type Ku80 (WT) or permutated Ku80 (K568R) treated with IR. Samples were harvested in time points as indicated. Three independent replications of WB experiments. β-actin was assayed to ensure equivalent loading and transfer

The Ku80 protein consists of three domains: an N-terminal von Willebrand A domain (vWA), a central DNA binding domain, and a divergent C-terminal domain (CTD).^31,32^ The Ku80 CTD, through which Ku80 interacts with DNA-PKcs proximal to its kinase domain, is present only in higher eukaryotes, and the K568cr site is in the CTD region of Ku80 (Fig. 4a). To precisely determine the role of K568 crotonylation on the Ku80 function, we performed a Co-IP assay. The results showed that the K568R mutation increases the binding of Ku80 to DNA-PKcs but doesn’t affect its binding to Ku70 (Fig. 4b). His pull-down assay outcomes illustrated that the K568R mutation increases the interaction within Ku80 and DNA-PKcs. However, the K568R mutation does not affect its binding with Ku70 (Fig. 4c).

To investigate whether K568R promotes the DNA-binding activity of Ku80, electrophoretic mobility change assay (EMSA) was performed. K568R enhances Ku80 DNA-binding activity compared to WT. In addition, in the presence of Ku70, the binding activity of Ku80 K568R to DNA increased more (Fig. 4d). To further evaluate the effect of K568 crotonylation on the binding ability of Ku80 and DNA-PKcs, we performed the molecular docking analysis of three-dimensional structure, using PIPER docking algorithm, on the interaction affinity of DNA-PKcs and WT Ku80 (K568) or K568-mutated Ku80 (K568R), K568-crotonylated Ku80 (K568Kcr). As shown in Fig. 4e, the PIPER pose energy score of the uncrotonylated Ku80 (K568) is lower than that of the crotonylated Ku80 (K568Kcr), suggesting that K568 site decrotonylation increases the binding affinity of Ku80 to DNA-PKcs. Moreover, the mutation of K568R, which lost the crotonylation site, has also a lower PIPER pose energy score than that of the K568Kcr Ku80. These data indicate that K568 decrotonylation of Ku80 protein has potential to affect the efficient interaction of Ku80 with DNA-PKcs.

To further validate the above results, we next analyzed the three-dimensional structure of crotonylated Ku80 and Ku80 K568R interacting with Ku70 (Supplementary Fig. 4f), Ku70 and DNA-PKcs (Supplementary Fig. 4g), and Ku70 and dsDNA (Supplementary Fig. 4h) compared to that of uncrotonylated WT Ku80 at the K568 site, respectively. As shown in the Supplementary Fig. 4f–h, the PIPER pose energy score of K568R Ku80, uncrotonylated WT Ku80, and crotonylated Ku80 are roughly equal when interacting with Ku70; the PIPER pose energy score of K568R Ku80 was lower than that of uncrotonylated WT Ku80 when interacting with Ku70 and DNA-PKcs or Ku70 and dsDNA; the PIPER pose energy fraction of crotonylated Ku80 was greater than uncrotonylated WT Ku80 when interacting with Ku70 and DNA-PKcs or Ku70 and dsDNA. These results indicated that the K568R mutation of Ku80 increased the binding of DNA-PKcs and DNA compared to that of the original uncrotonylated WT Ku80 and that inhibition of Ku80 crotonylation affected the combination of Ku80 to DNA-PKcs.

**Fig. 5:**
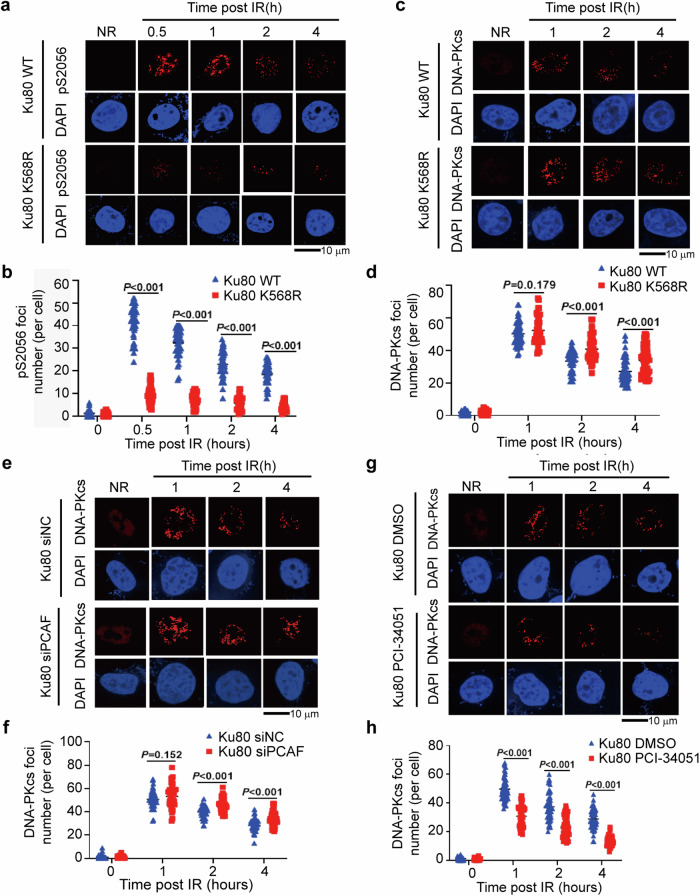
K568R permutation of Ku80 inactivates DNA-PKcs S2056 autophosphorylation and attenuates DNA-PKcs dissociation from DNA damage. **a** Immunofluorescence (IF) staining of DNA-PKcs pS2056 was performed in stable cell lines bearing exogenous wild-type Ku80 (WT) or mutant Ku80 (K568R) treated with or without IR. **b** Quantification of DNA-PKcs pS2056 foci formation. 50 cells were quantified and analyzed. **c** Effect of Ku80 K568R permutation on DNA-PKcs foci formation in irradiated cells. Immunofluorescence (IF) staining of DNA-PKcs was performed in stable cell lines bearing exogenous wild-type Ku80 (WT) or permutated Ku80 (K568R) treated with IR. **d** Quantification of DNA-PKcs foci formation affected by K568R permutation. 50 cells were quantified and analyzed. **e** Effect of PCAF depression on DNA-PKcs foci formation in irradiated cells. Stable cell lines bearing exogenous wild-type Ku80 (WT) were transfected with PCAF-specific siRNA (siPCAF) or control siRNA (siNC) and treated with IR or not, then IF assay was performed with DNA-PKcs antibody. **f** Quantification of DNA-PKcs foci formation affected by depressing PCAF. 50 cells were quantified and analyzed. **g** Effect of HDAC8-specific inhibitor PCI-34051 on DNA-PKcs foci formation in irradiated cells. The cells were treated with HDAC8-specific inhibitor PCI-34051 (10 μM, 6 h) or DMSO solvent as control, and DNA-PKcs foci were observed by IF assay after IR. **h** Quantification of DNA-PKcs foci formation affected PCI-34051. 50 cells in each group were quantified and analyzed. DAPI was used to be nuclear staining. Data are represented as mean ± SD. A *p*-value less than 0.05 indicates a significant difference

DNA-PKcs phosphorylation is the key event in determining its activity in NHEJ pathway of DSB damage repair. We tested the influence of the Ku80 K568R mutation on DNA-PKcs S2056 and T2609 autophosphorylation. Although the K568R mutation affected the interaction between Ku80 and DNA-PKcs, it dramatically attenuated the irradiation-induced DNA-PKcs autophosphorylation at S2056 (Fig. 4f). Furthermore, the K568R mutation did not affect the phosphorylation dynamic of DNA-PKcs at T2609 (Supplementary Fig. 4i). Immunofluorescence staining (IF) assay further indicated that the K568R mutation impacts the formation of DNA-PKcs pS2056 foci in irradiated cells (Fig. 5a, b). K568 point mutation results in disrupting DNA-PKcs-S2056 autophosphorylation in the condition of exposure to DNA damage. These outcomes suggested that Ku80 K568 site and its PTM status may impact the interaction of Ku80 with DNA-PKcs, and DNA-PKcs activation and repair function.

It is believed that DNA-PKcs should dissociate from the damage site after accomplishing its task of initiating DNA DSBs repair processing, to make room for the subsequent repair proteins. To verify whether Ku80 crotonylation affects the dissociation of the DNA-PK complex after DNA damage repair, IF assays were conducted to evaluate the formation of DNA-PKcs foci. Although the formation of DNA-PKcs-pS2056 foci is deficient (Fig. 5a, b), the K568R mutation affected the duration of DNA-PKcs foci in irradiated cells (Fig. 5c, d). However, in the cells in which PCAF was knocked down by siRNA to inhibit Ku80 crotonylation, the dissociation rate of DNA-PKcs foci was slowed after DNA damage (Fig. 5e, f). Meanwhile, the HDAC8-specific inhibitor PCI-34051, which prevents decrotonylation of Ku80 K568cr, was observed to affect the disappearance of DNA-PKcs foci (Fig. 5g, h). Notably, HDAC inhibition leads to an increase in Ku80 K568Kcr levels, which in turn reduces the interaction between Ku80 and DNA PKcs. This effect was supported by previous Uematsu et al. study that the result of Ku80 is essential for recruitment of DNA-PKcs at DSBs site.^33^ Together, these findings indicated that the declined initial level of DNA-PKcs in DSBs sites by HDAC8 inhibitor could be attributed to the impacted recruitment of DNA-PKcs at DSBs sites. The status of K568 site crotonylation may have potential for the functional regulation of Ku80-DNA-PKcs in DNA DSB repair, and we next clarified how K568 influences DNA-PKcs autophosphorylation.

### Decrotonylation of Ku80 K568cr is required for the subsequent CBX4-mediated SUMOylation at the same site for the activation of DNA-PKcs upon DNA damage

**Fig. 6:**
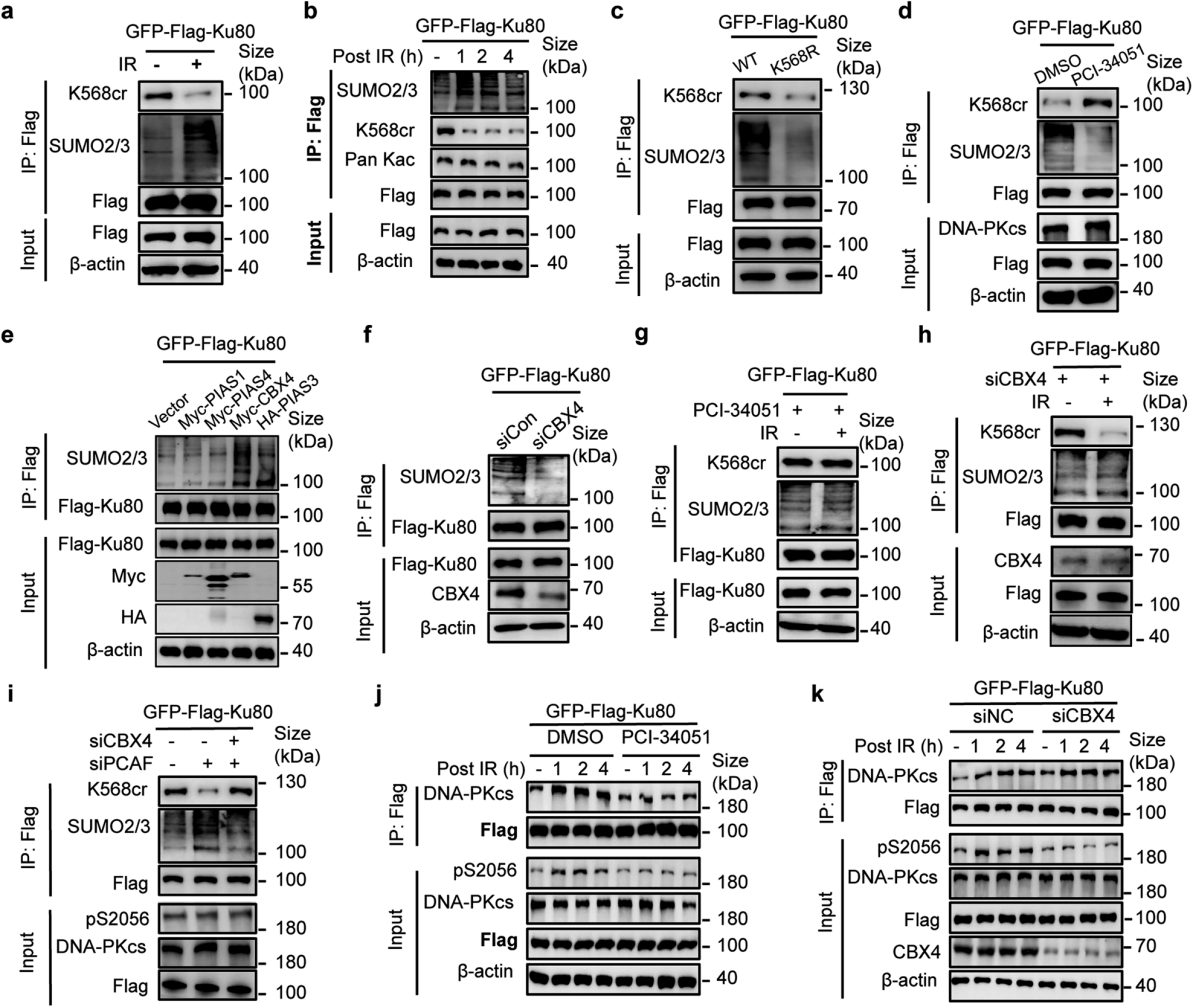
PTM switching of Ku80 K568 from crotonylation to SUMOylation to activate autophosphorylation of DNA-PKcs S2056 in response to DNA damage. **a** IP assay to measure level changes of crotonylation and SUMOylation of Ku80 in the cells irradiated with 8 Gy γ-ray. **b** IP analysis of Ku80 SUMOylation and K568 crotonylation dynamic in the cells at different times post-8Gy irradiation. **c** Determination of Crotonylation and SUMOylation by IP assay in the cells with exogenous wild-type Ku80 (WT) or mutant Ku80 (K568R). **d** Effects of HDAC8-specific inhibitor PCI-34051 on crotonylation and SUMOylation of Ku80. GFP-Flag-tagged Ku80 immunoprecitated by Flag antibody from the cells treated with PCI-34051 (10 μM, 6 h) or DMSO for control was analyzed by immunoblotting with the indicated antibodies. **e** Identification of E3 ligase for Ku80 SUMOylation. The cells were transfected with a series of indicated E3 ligases’ plasmids, respectively; the Ku80 SUMOylation levels were examined by IP and immunoblotting. **f** Decrease of Ku80 SUMOylation by suppressing CBX4. The SUMOylation level of GFP-Flag-tagged Ku80 was analyzed by IP coupling immunoblotting with the indicated antibodies in the cells with or without siRNA-mediated CBX4 knockdown. **g** Ku80 SUMOylation was assessed using HDAC8-specific inhibitor PCI-34051 with or without radiation treatment. The crotonylation and SUMOylation of Ku80 were analyzed by immunoblotting with the indicated antibodies after irradiation in cells pretreated with PCI-34051 (10 μM) for 6 h. **h** Depression of CBX4 blocks Ku80 SUMOylation induced by irradiation. CBX4 was knocked down by siRNA, and the crotonylation and SUMOylation of Ku80 in irradiated cells were analyzed by immunoblotting with the indicated antibodies. **i** IP and western blotting were performed with the indicated antibodies in cells with PCAF or CBX4 knockdown by siRNA. **j** Effect of HDAC8-specific inhibitor PCI-34051 on IR-induced DNA-PKcs Ser2056 phosphorylation. Stable cell lines bearing exogenous GFP-Flag-Ku80 were treated with PCI-34051 (10 μM) for 6 h or with DMSO for control, and DNA-PKcs Ser2056 phosphorylation was subsequently evaluated by Western blotting after IR. **k** Effect of CBX4 depression on IR-induced DNA-PKcs Ser2056 phosphorylation. CBX4 siRNA-depleted HeLa cells were treated with or without IR, and DNA-PKcs Ser2056 phosphorylation was examined by western blotting. Three independent replications of WB experiments. β-actin was assayed to ensure equivalent loading and transfer

It has been reported that Ku80 potentially undergoes SUMOylation by SUMO2/3 at several lysine residues,^20,34–36^ but the SUMOylation site(s), dynamic and the role are not clear. We speculated that SUMOylation of Ku80 is involved in its regulation on DNA-PKcs kinase activity during DNA damage response through coregulation with crotonylation. To validate the SUMOylation of Ku80 and cross-talk with Ku80 crotonylation on the same lysine residue, we first evaluated the effect of IR on total Ku80 SUMOylation levels. Intriguingly, IR decreased the level of Ku80 K568cr, as expected, and simultaneously increased SUMOylation of the Ku80 (WT) protein (Fig. 6a). Ku80 SUMOylation increased post-IR while comparing with non-IR, meanwhile the acetylation level (Kac) did not change at the observed time points after IR (Fig. 6b). Figure 6c further demonstrates that the K568R mutation affected both the SUMOylation and crotonylation of Ku80. Moreover, the treatment of HDAC8-specific inhibitor PCI-34051 upregulated Ku80 K568 crotonylation and simultaneously led to downregulation of Ku80 SUMOylation in cells (Fig. 6d), indicating that at the K568 residue of Ku80, both of SUMOylation and crotonylation occurred. Collectively, these findings indicate that upon DNA damage by radiation, Ku80 K568 crotonylation is replaced by SUMOylation, at this site the crotonylation and SUMOylation could be mutual influence in regulation of DNA-PK function.

To explore the SUMO E3 ligase for Ku80 SUMOylation, we tried to transfect cells using Myc-tagged SUMO E3 ligases, such as PIAS1, PIAS4, CBX4, and HA-tagged PIAS3. It was found that only CBX4 dramatically promoted Ku80 SUMOylation (Fig. 6e). Moreover, there was an interaction between CBX4 and Ku80 (Supplementary Fig. 5a, b). SiRNA-mediated CBX4 knockdown in cells results in significantly reduced Ku80 SUMOylation under normal conditions (Fig. 6f). This result further indicates that CBX4 could mediate Ku80 SUMOylation. The HDAC8-specific inhibitor PCI-34051 abrogates the IR-induced downregulation of Ku80 K568cr, consequently, resulting in blockage of radiation-induced Ku80 SUMOylation in the cells (Fig. 6g). These results indicate that decrotonylation of Ku80 K568cr by HDAC8 is a prerequisite for subsequent SUMOylation at this site following DNA damage. In cells in which CBX4 expression was knocked down by siRNA, Ku80 SUMOylation was not increased following IR (Fig. 6h), suggesting that CBX4 is responsible for Ku80 SUMOylation upon DNA damage. However, siRNA-mediated knockdown of CBX4 does not influence the reduction of Ku80 K568cr level mediated by irradiation (Fig. 6h), suggesting that there is the notion of crosstalk between the SUMOylation and crotonylation of Ku80 at K568. To explore whether Ku80 SUMOylation and crotonylation affect DNA-PKcs S2056 autophosphorylation, we performed immunoprecipitation (IP) analysis in cells treated with or without radiation. Under the normal growing conditions, depressing PCAF alone or together with CBX depression does not change the DNA-PKcs S2056 autophosphorylation level (Fig. 6i, Supplementary Fig. 5c). However, the DNA-PKcs S2056 autophosphorylation induced by radiation decreased after blocking Ku80 decrotonylation through treatment of HDAC8-specific inhibitor PCI-34051 in irradiated cells, the interaction was also declined between Ku80 and DNA-PKcs upon DNA damage (Fig. 6j). Furthermore, knockdown CBX4 resulted in deficiency of IR-induced DNA-PKcs S2056 autophosphorylation, indicating inhibition of Ku80 SUMOylation did not affect the irradiation-increased binding of Ku80 to DNA-PKcs but influenced DNA-PKcs S2056 autophosphorylation (Fig. 6f, k). Taken together, these results indicate that when cells encounter DNA damage induced by radiation, HDAC8 mediates decrotonylation of Ku80 K568cr, which affects the interaction of Ku80 with DNA-PKcs. Consequently, the K568 site is released for SUMOylation subsequently to facilitate DNA-PKcs S2056 phosphorylation.

### The Ku80 K568R mutation impacts DNA DSB repair and sensitizes tumors to radiotherapy

**Fig. 7:**
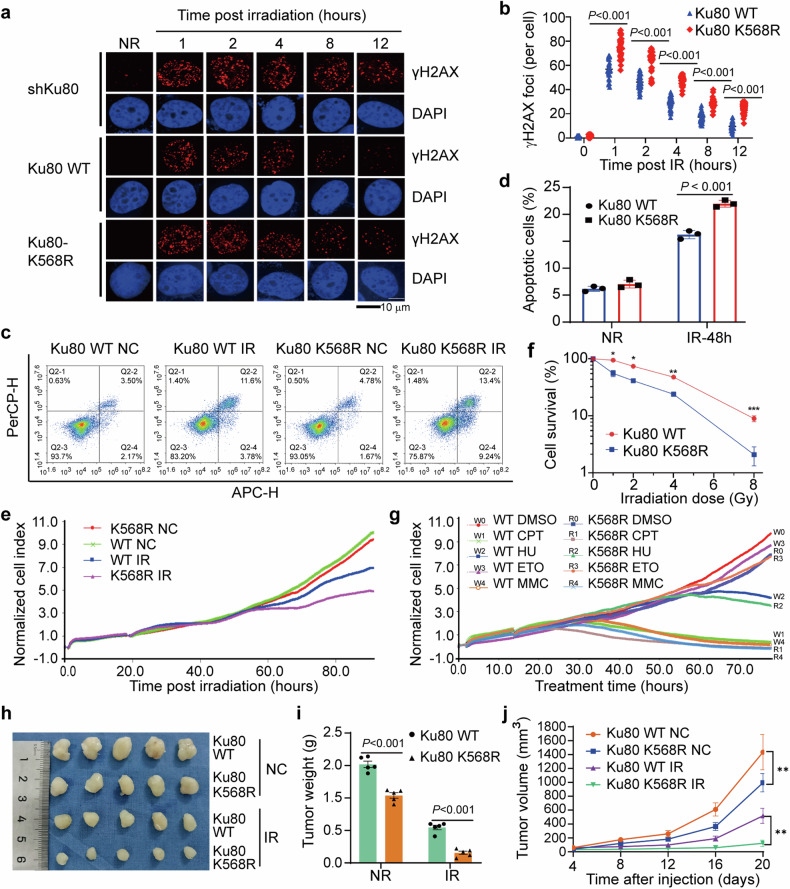
K568R mutation of Ku80 sensitizes cancer cells to DNA damage agents and inhibits cancer development. **a** The Ku80 K568R mutation decreases the efficiency of DNA DSB repair, as shown by the increase in the number of residual γ-H2AX foci after 8 Gy irradiation. DAPI was used to be nuclear staining. **b** The γ-H2AX foci in 50 cells were quantified and analyzed. A *p*-value less than 0.05 indicates a significant difference; two-tailed Student’s t-test. **c** Flow cytometric histograms of apoptosis detection. Ku80 WT and K568R mutant HeLa cells were irradiated with 8 Gy of γ-ray. Apoptosis was detected at 48 h after irradiation. **d** Quantification of apoptosis induction was performed. The data are the means ± SDs from three independent experiments. *P* < 0.001, comparison between the wild-type group and the Ku80 WT group. **e** Growth curves of Ku80 WT and Ku80 K568R mutant HeLa cells generated using the xCELLigence Real-Time Cell Analyzer (RTCA)-MP system. **f** Survival of Ku80 WT and K568R mutant HeLa cells exposed to IR. The data are the means ± SDs from three independent experiments. *p < 0.1, **p < 0.01, ***p < 0.001. **g** The sensitivity of Ku80 WT and Ku80 K568R mutant HeLa cells to DNA damage or replication stress-inducing agents was determined by the xCELLigence Real-Time Cell Analyzer (RTCA)-MP system. **h**, **i**, **j** Tumorigenicity of Ku80 WT and Ku80 K568R mutant HeLa cells in nude mice. Ku80 WT (0.1 ml; 1 × 10^6^ cells) or Ku80 K568R mutant HeLa cells were injected into each nude mouse (n = 5 in each group). In addition, we list the treatment details in the Materials and Methods in available supplementary file. Tumor growth condition was recorded in every 3 days. The weight of tumor tissues were measured after sacrificing the mice. Data are presented as means ± SDs. A *p*-value less than 0.05 indicates a significant difference. **p < 0.01. β-actin was assayed to ensure equivalent loading and transfer

Our results demonstrate that Ku80 K568 and its PTM status is critical for the functional regulation of Ku80 and DNA-PK complex. HDAC8-mediated Ku80 K568cr decrotonylation exposes K568 for the SUMOylation mediated by CBX4 and subsequent DNA-PKcs activation and initiating DNA DSBs repair. Using immunofluorescence staining, we detected the formation of IR-induced γ-H2AX foci, a molecular marker of DSBs. Much more γ-H2AX foci were detected in cells expressing mutant Ku80 K568R than in those expressing WT Ku80 after irradiation (Fig. 7a, b), suggesting that the Ku80 K568R mutation leads to deficiency of DNA DSB repair. Western blotting analysis also revealed elevated residual levels of γ-H2AX in Ku80 K568R mutant cells post-irradiation (Supplementary Fig. 6a). We also investigated whether the Ku80 K568R mutation affects apoptosis induction in cancer cells post exposing radiation. We found that the percentage of apoptotic Ku80 K568R mutant cancer cells greatly elevated post irradiation (Fig. 7c, d). We also observed that the Ku80 K568R mutation would impact the sensitivity of cancer cells to irradiation, as indicated by the decreases in cell growth (Fig. 7e) and colony formation (Fig. 7f). Moreover, the Ku80 K568R mutation also sensitizes cancer cells to other DNA-damaging agents, including HU, CPT, ETO, and MMC (Fig. 7g). Finally, using tumor-harboring nude mice, we found the growth rate of tumors originated from Ku80 K568R mutant cancer cells decreased, moreover, the tumors were much more sensitive to chemo-radiotherapy (Fig. 7h–j). Collectively, these findings indicate that Ku80 K568 is a critical site for its function in initiating DNA DSB repair and determining sensitivities of cancer to chemo-/radiotherapy.

### Effects of Ku80 K568R on NHEJ and radioresistance are more broadly applicable across different cancers

Above findings have showed that the Ku80 K568 impacts DNA DSB repair and sensitizes tumors to radiotherapy. To explore whether its effects were applicable across different cancers, we used A549, HepG2, U87 cancer cells to further validate our findings. As shown in Supplementary Fig. 7a–f, Ku80 K568 crotonylation decreased but SUMOylation increased after radiation treatment in additional A549, HepG2, U87 cancer cells, indicating the effect of radiation on Ku80 K568 crotonylation and SUMOylation may occur in multiple cancer cells. Furthermore, we have observed previously that post irradiation, the Kcr of Ku80 decreased and the corresponding SUMOylation increased. When HDAC8 inhibitor, PCI-34051 was administrated in A549, HepG2, U87 cancer cells, the radiation-induced changes of Kcr of Ku80 decreased and the corresponding SUMOylation increased were blocked, further indicating that HDAC8-mediated decrotonylation is necessary for the radiation-induced SUMOylation of Ku80 (Supplementary Fig 8a–f). Therefore, if the K568cr of Ku80 does not change after ionizing radiation, and lysine cannot be allowed to undergo SUMO modification, resulting in no change in the SUMO modification of Ku80 after ionizing radiation. This result further verified that Ku80 decrotonylation modification is a prerequisite for SUMO modification to occur. We then questioned whether PCI-34051 could affect DNA DSBs repair across different cancers. The results showed that compared with non-radiation, more γ-H2AX foci were observed in PCI-34051 and radiation treated A549 cancer cells (Supplementary Fig. 9), HepG2 cancer cells (Supplementary Fig. 10), U87 cells (Supplementary Fig. 11), and HeLa cells (Supplementary Fig. 12) and MCF-7 cells (Supplementary Fig. 13), indicating that decrotonylation of Ku80 K568cr is required for activation of DNA DSB repair.

As of Fig. 4a, d showed Ku80 has a DNA binding region, to explain whether Ku80 binds to DNA alone, we repeated Fig. 4d experiment and found the ability of Ku80 binding to DNA alone is weak, however, the addition of Ku70 may increase the binding affinity (Supplementary Fig. 14a–d). This finding is consistence with Wang et al. report that purified Ku80 does not bind to DNA alone.^37^ As report as well as Choon Kit Tang et al. wrote in the book of *Biological DNA Sensor*, 2014, in eukaryotic cells, Ku proteins comprises of 3 domains, one is an N-terminal von Willebrand A domain (vWA), another is a central DNA binding domain, the third is a diverged C-terminal domain (CTD) (Supplementary Fig. 14e),^38^ it needs further more investigation to explore the binding activity of Ku80 regions with DNA. In addition, based on the finding that Ku80 lysine crotonylation facilitates NHEJ, we further showed that NHEJ efficiency was directly affected in shKu80 cells transfected with the point mutant Ku80 K568R plasmids as comparison with Ku80 WT plasmids (Supplementary Fig. 15). Moreover, to provide more data to support the contention that the K568R mutant impacts DNA repair, we performed the immunofluorescence staining of DNA-PKcs and neutral comet assays. Compared to cells expressing the exogenous Ku80 WT, the cells expressing the mutant Ku80 K568R increased the residual DNA-PKcs foci with a post-IR time-dependent response. Neutral comet assay further identified that tail moments were much more in cells treated with mutant Ku80 K568R than that of treated with Ku80 WT(Supplementary Fig. 16a–c). These results further indicated that Ku80 K568R affects DNA damage repair.

In addition, we provided the sensitivity of Ku80 K568R mutant cells to DNA damage or replication stress-inducing agents at 72 h based on the Fig. 7e results. It clearly showed that compared to DMSO treatment, Ku80 K568R mutant HeLa cells had decreased normalized cell index after treating with ETO (Supplementary Fig. 17a), CPT (Supplementary Fig. 17b), HU (Supplementary Fig. 17c), and MMC (Supplementary Fig. 17d), indicating Ku80 K568R may affect chemotherapy, showing there exist important potential of Ku80 K568 in clinic chemo-and radio-therapy. We also calculated the tumor doubling time of Fig. 7f. Notably, radiation combined with Ku80 K568R led to an increase in tumor doubling time, indicating Ku80 K568R enhances the antitumor effect of radiation (Supplementary Fig. 18, Table 2). Moreover, we provided evidence showing that Ku80 K568cr decreased in nucleus but the Ku80 SUMO2/3 increased after treatment of radiation (Supplementary Fig. 19). Interestingly, using plasmids of Flag-Ku80 S577A and Flag-Ku80 S580A mutants, we further found that the phosphorylation at S577 and S580 of Ku80 near K568 did not affect crotonylation and subsequent SUMOylation (Supplementary Fig. 20a–d). This indicates that the regulation function of Ku80 K568 may not be affected by its’ nearby phosphorylations.

To further substantiate the clinical relevance of our findings, we performed patient-derived xenograft (PDX) models. Lung cancer and meningioma patient-derived xenografts were transplanted into 5-week-old male BALB/c-nude mice. As shown in Supplementary Fig. 21, compared with the mice inoculated with the treatment of radiation or PCI-34051 alone, the combination of radiation and PCI-34051 showed more intense reduction trend in tumor volume and tumor weight, indicating Ku80 K568 affects patient-derived cancer xenografts and confers tumor radiation resistance. Moreover, a shorter timeline assay within 60 minutes after irradiation further ascertained the kinetics of decronylation (Supplementary Fig. 22). Finally, the colony formation assay demonstrated that the colony forming ability and survival fractions were much more declined for the Ku80 K568R mutated cells exposed to different DNA damage anticancer agents as compared with the Ku80 wild-type cells (Supplementary Fig. 23a–c).

## Discussion

Efficient repair of damaged DNA is critical for the maintenance of genomic integrity and fidelity of genetic information. In reverse, increased DNA repair capability in cancer cells contributes to chemo-/radioresistance of malignant tumors. DNA-PK complex assembly and activation at the DNA break-ends is precisely the hallmark of DNA repair initiation. In this study, we uncovered that Ku80 PTM switching at the K568 site from crotonylation to SUMOylation is the critical molecular event of driving DNA-PK assembly and activation for efficiently initiating DNA repair. Previous studies have suggested that lysine crotonylation is involved in DSB repair.^26–28^ The precise mechanism that the effect of Lys crotonylation and sumolyation on NHEJ in the DDR remains unclear. This study characterized the global crotonylome under IR-induced DNA damage stress. We identified 20910 Kcr sites across 3844 proteins, which may be the largest crotonylome dataset currently.^21,27,39,40^ From the perspective of radiotherapy, this crotonylome dataset not only enlarges the Kcr proteome related databases but also increases the understanding of cellular physiological alterations and functions in protein crotonylation under the status of DNA damage response. DNA double-strand break is a crucial DNA lesion induced by IR, and at least 16 proteins in DSB repair pathways were crotonylated at 1–78 lysine sites according to LC‒MS/MS analysis. Ku80, DNA-PKcs, exhibited altered crotonylation following IR exposure. KEGG analysis of the altered crotonylome data demonstrated that the NHEJ pathway of DSB repair, is an enriched functional pathway of the differentially expressed Kcr proteins, confirming that Kcr proteins participate in the process of DNA damage repair.

The Ku70/K80 heterodimer is an NHEJ-initiating factor that binds to the ends of DNA DSBs and facilitates the recruitment of DNA repair proteins, including DNA-PKcs.^2,3,6,41^ Ubiquitination and deubiquitylation of Ku80 are important for Ku removal from DSBs at the end of the NHEJ process.^18,19^ In this work, we found that HDAC8-mediated decrotonylation of Ku80 at K568 upon DNA damage affected Ku80 K568R binding to DNA-PKcs but impacted its activation. These results imply that the role of Ku80 crotonylation differs from that of ubiquitination and deubiquitination and that Ku80 crotonylation plays a distinct role in DNA-PK complex assembly and activation. K568 is in the CTR region of Ku80 at the DNA binding border.^31,32^ Previous studies have reported that knockout of Ku80 in human cells resulted in lethality.^42,43^ In this study, although we generated Ku80 knockdown cancer cells, this could be a hybrid clone instead of monoclonal. In addition, we generated cells harbored the K568R mutant and the K338-K568R double mutant and found that the signal of Pan-Kcr only decreased in the cells with the K568R mutated Ku80, but the crotonylation is not completely lost. It has been reported that Ku80 can be phosphorylated,^44^ sumoylated^45^, and ubiquitinated.^46^ Our study demonstrated that other Ku80 crotonylation sites may exist, which need to be further investigated to explore why the crotonylation is not entirely lost in the K568R or K338R K568R cell line. Through EMSA experiments, we found that compared to the WT, the Ku80 K568R mutation enhances the binding of Ku80 to DNA, which is observed in the presence or absence of Ku70. These results suggest that the Ku80 K568 mutation or crotonylation status of K568cr affects the formation of the DNA-PK holoenzyme complex, while K568 mutation may result in other critical events that could abolish DNA-PK complex function or activity in DSB repair.

The formation of the DNA-PK holoenzyme complex can mediate the phosphorylation of DNA-PKcs and phosphorylation of other NHEJ factors.^47,48^ However, it is still unclear how DNA-PKcs autophosphorylation is activated. We found that the Ku80 K568R mutation leads to reduced autophosphorylation of DNA-PKcs S2056 and pS2056 foci formation, as well as NHEJ activity. These results imply that the K568 site is important for Ku80 function and that other modifications or molecular event(s) occur at the K568 site to dynamically co-regulate the function of Ku80 and the DNA-PK complex in DSB repair. Crosstalk indicates that proteins are modified by multiple PTMs and that these PTMs can interact with each other. Due to the lysine residues may be bind with different PTMs, the crosstalk among various PTMs would be supposed to be competitive. Importantly, Kcr is typically similar to Kac, which is another kind of protein modification occurs on the e-amino position of lysine.^23,29,49–51^ However, we found that the Ku80 acetylation level did not change after IR or after Ku80 K568R expression, indicating that the Ku80 K568 site is not acetylated. Therefore, the K568 site is functionally essential for Ku80, and the crotonylation is a critical and dynamically changed PTM on Ku80 in responding to IR exposure.

Some studies quantifying endogenous SUMO modifications have reported that Ku80 can undergo SUMOylation by SUMO2/3 at several sites, including K568.^34–36^ Recently, Dan Feng et al. reported that SUMO2/3-mediated Ku80 SUMOylation occurs at K307, K568, and K285 and that Ku80-K307 SUMOylation promotes oxaliplatin resistance in CRC cells.^20^ In our work, we also verified Ku80 K568 SUMOylation and revealed that Ku80 crotonylation antagonizes SUMOylation at the K568 residue in cells under normal growth conditions. Upon IR-induced DNA damage, Ku80 K568cr is decrotonylated and subsequently SUMOylated by CBX4. This dynamic switch of the Ku80 PTM from crotonylation to SUMOylation may have potential to affect the interaction between Ku80 and DNA-PKcs and activation of the DNA-PK complex.

IR-induced DNA damage can trigger Ku80 PTM switching, and HDAC8-mediated decrotonylation is the critical step in this process. We found that the HDAC8-specific inhibitor PCI-34051 increases the Ku80 K568cr level and decreases Ku80 SUMOylation in cells. SUMOylation is critical for orchestrating DNA repair and affects protein recruitment, translocation, localization, stability, and kinase activity.^16,52–55^ SUMOylation has been detected in many DNA damage response-related proteins, including Ku70.^16,53–55^ Although it has been reported that Ku80 can be SUMO-modified, the enzymes it is regulated by and the regulatory mechanism involved in the DDR process are still unclear. Our data showed that DNA-PKcs-pS2056 phosphorylation was inhibited and that Ku80 binding to DNA-PKcs was not affected by siRNA-mediated CBX4 knockdown under DNA damage conditions. This result further demonstrated that Ku80 K568cr decrotonylation is required for its interaction with DNA-PKcs and that SUMOylation of K568 promotes DNA-PKcs-pS2056 activation during DNA damage. In Ku80 K568R mutant cells, DNA-PKcs S2056 phosphorylation aberrantly reduces, resulting in NHEJ inhibition.

**Fig. 8:**
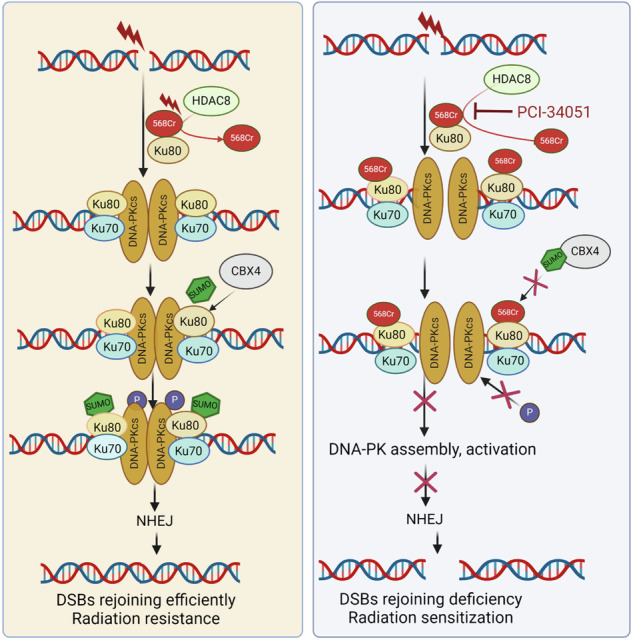
Proposed model for PTM conversion of Ku80 K568 from crotonylation to SUMOylation to control DNA DSBs repair and radiosensitivity. The crotonylation of Ku80 Lysine 568 (K568cr), mediated by PCAF, is decrotonylated by HDAC8 upon DNA damage, which fosters the interaction of Ku80 and DNA-PKcs and also empties the room for the subsequent SUMOylation by CBX4 E3 ligase at the same site. Failure of K568cr decrotonylation hinders Ku80 SUMOylation. Ku80 K568 SUMOylation is obligated for activating DNA-PKcs S2056 autophosphorylation to promote DNA double-strand break repair. Prevention of K568cr decrotonylation by HDAC8-specific inhibitor PCI-34051, or blockage of K568 SUMOylation by suppressing CBX4 leads to DNA-PK inactivation and defective DNA DSBs repair, which can sensitize cancer cells to radiotherapy

NHEJ is a major pathway of DSBs repair. Damage site recruitment and activation of DNA-PK complex is the critical event for NHEJ initiation^56^ DNA-PK is important for determining the activity of NHEJ.^4,7,57^ Ku80 K568R mutation (abrogated the Kcr and SUMOylation site) affected the interaction between Ku80 and DNA-PKcs to affect DNA-PK complex formation and activity at DSBs. These data further indicate that the dynamic PTM switching of Ku80 K568 may be important for precisely targeting radiation resistance through regulation of DSBs rejoining efficient-related pathways (Fig. 8).

Indeed, the Ku80 K568 site is critical for its role in DNA-PK assembly and activation via the dynamic switching of PTM from crotonylation to SUMOylation in the initiation of DNA DSBs repair, but further studies are needed to develop the specific chemical inhibitors for this PTM switching based on the precise structure assay. This kind of inhibitor may provide promising new strategy to overcome the radio- or chemo-resistance of cancers. It would be valuable to determine the foci formation of SUMOylated Ku80 K568 upon DNA damage and confirm the influence of blocking decrotonylation of K568cr. Due to a lack of specific antibodies, we have not conducted this experiment. Finally, although we developed a Ku80 K568cr-specific antibody, we have not yet screened human tumor samples (TMA) and normal tissues using this antibody to evaluate endogenous levels of Ku80 crtononylation and correlate with patient survival or outcome. In addition, the PIPER pose energy scores are simply predictions and not actual experiments. Therefore, it will be necessary to further evaluate the clinical significance of these findings in additional cellular background, including models of clinical diseases.

In summary, our findings reveal a novel mechanism driving DNA-PK assembly and DNA DSBs repair activation through the dynamic conversion of the Ku80 K568 PTM from crotonylation to SUMOylation. We speculate that this mechanism model of PTM conversion might also be fundamental to other biological processes, playing a critical role in controlling the associated cellular events. Such PTM conversion of functional dominant provides a new strategy for developing targeted therapies for diseases, including cancer.

## Materials and methods

The animal experiments were performed in accordance with the Laboratory Animal Guidelines of Welfare and Ethics. Animal experiment of xenograft was approved by the Animal Care and Use Committee of the Academy of Military Medical Sciences (IACUC-DWZX-2024-529). This study collected cancer samples from the human subjects. Patient-derived tumors were obtained from Xiangya Hospital, Central South University, informed consent was obtained from the participants before the study and was approved by the Xiangya School of Public Health Central South University Medical Ethics Committee (XYGW-2022-56).

### Cell lines, culture, and transfection

In this study, human HEK-293T, MCF-7, A549, HepG2, and HeLa cell lines were used. These cell lines were all purchased from the American Type Culture Collection (ATCC). The cultivated conditions we used for these cell lines were the below: 10% (v/v) FBS-DMEM (Dulbecco’s modified Eagle’s medium) supplemented with 1% (v/v) penicillin–streptomycin. The selection of MCF-7 and HeLa cell lines was due to they were used to investigation of chemotherapy and radiotherapy responses in common in previous reports.^58,59^ The selection of HEK-293T was due to it comes from human embryonic kidney cells, which rarely express endogenous receptors required for extracellular ligands and is relatively easy to transfect. HEK-293T is a commonly used cell line for studying the expression of exogenous genes.^60^ Human U87 cell also was purchased from the American Type Culture Collection (ATCC). The cultivated conditions were selected with 10% (v/v) FBS-MEM (Minimum Essential Medium) supplemented with 1% (v/v) penicillin–streptomycin and 1% NEAA (MEM Non-Essontial Amino Acids). Human shKu80 HeLa cells, GFP-Flag-Ku80 (WT) HeLa-Ku80 KO cells, and GFP-Flag-Ku80 (K568R) HeLa-Ku80 KO cells were generated using CRISPR–Cas9 technology and were cultivated in 10% (v/v) FBS-DMEM (Dulbecco’s modified Eagle’s medium) supplemented with 1% (v/v) penicillin–streptomycin, 15 µg/mL blasticidin, 400 µg/mL G418 sulfate, and 0.5 µg/mL puromycin. For cell maintenance, a humid chamber/incubator at 37 °C with 5% CO_2_ was utilized. All transfections were performed using Lipofectamine 2000 (Invitrogen) or Lipofectamine 3000 (Invitrogen) according to the manufacturer’s instructions.

### Computational prediction of protein structure

Download the complex structure with PDB ID: 7Z87 from the PDB database, and extract the PDB structures of DNA-PKcs (7Z87, Chain A) and dsDNA (7Z87, Chain D,E) protein; Download the AlphaFold structures of XRCC5 (P13010, Ku80) and XRCC6 (P12956, Ku70) from the UniProt database, perform crotonylation modification at XRCC5 K568, obtain modified XRCC5 and XRCC6 proteins, and preprocess these docking structures (fill in missing amino acid residues, fill in missing hydrogen atoms); Perform molecular docking of the wild-type/modified XRCC5 with XRCC6 using ClusPro 2.0 (https://cluspro.bu.edu/, default parameters) to obtain the binding poses of the complex; Save the PDB structure files and the binding pose energyto quantify the binding affinity between XRCC5 and XRCC6. Obtain the XRCC5-XRCC6 complex. Then, dock the complex with DNA-PKcs and dsDNA separately to obtain the binding poses.

### Chromatin fractions extraction

Chromatin extraction is performed according to the instructions of the chromatin extraction kit. Cells (1 × 10^6^) were gently lysed with 200 µL Working Lysis Buffer (1×) for 10 min on ice. After vortex vigorously for 10 s and centrifuge at 5000 rpm for 5 min, the supernatants were soluble fractions. The remaining parts were washed with Working Extraction Buffer to chromatin pellet and resuspend by carefully pipetting up and down (50 µL/1 × 10^6^cells). Next, the sample was incubated on ice for 10 min and sonicated 2 × 20 s to increase chromatin extraction. After centrifuge at 12,000 rpm at 4 °C for 10 min, the sample was added to Chromatin Buffer at a 1:1 ratio. The soluble and chromatin fractions were analyzed by SDS-PAGE and immunoblotting.

### Neutral comet assays

GFP-Flag-Ku80 (WT), GFP-Flag-Ku80 (K568R) HeLa-Ku80 KO cells were plated into 6-well plates at a concentration of 1 × 10^5^ cells per well. After treatment with IR (8 Gy), cells were harvested at the indicated time and then utilized for Comet assays according to the manufacturer’s instructions. In summary, the cells were embedded in low-melting-point agarose on a slide and treated with lysis buffer for 1 h at 4 °C. The slides were then soaked in 1× electrophoresis buffer for ~40 min to denature the DNA. After that, electrophoresis was conducted for 25 min at 25 V. Following electrophoresis, the slide was stained with propidium iodide (PI) and a cover-glass was placed on top. To quantify the results, the comets on each gel were examined under a fluorescence microscope, and the tail length and tail moment were recorded to assess the level of DNA damage.

Other experiments’ information is available in supplementary file. These experiments include Co-IP, western blotting and nucleoplasmic isolation experiment, Crotonylation modification omics assay, In vitro crotonylation and decrotonylation assay, Electrophoretic mobility shift assay (EMSA), His-pull down assay, NHEJ DNA repair assay, Immunofluorescence assay, Laser micro-irradiation assay, Clony forming assay Proliferation and cell viability assays, Flow cytometry, Xenograft experiment, and PDX (Patient-derived tumor xenograft) models.

### Quantification and statistical analysis

Here, in this study, we used IBM SPSS 23.0 or GraphPad Prism software 8.02 to do analysis. For two groups’ statistical analysis, two-tailed unpaired Student’s t-test was used. For multiple groups’ statistical analysis, ONE-Way ANOVA was used. Means ± SDs are listed in the results and figures unless otherwise indicated. *p < 0.05, **p < 0.01, ***p < 0.001 indicates statistical significance, and the statistical details are shown in the figures and figure legends.

## Supplementary information

Supplementary File

## Data Availability

The mass spectrometry proteomics data have been deposited to the ProteomeXchange Consortium via the PRIDE (website: https://www.ebi.ac.uk/pride/) partner repository with the dataset identifier PXD061039. All data needed to evaluate the conclusions in the article are present in the article and/or the Supplementary Materials. The data informing the results of this article will be shared upon reasonable request to the corresponding author. This paper does not report the original code.
