# Supplementary material for: Conversion of Ku80 K568 crotonylation to SUMOylation facilitates DNA non-homologous end joining and cancer radioresistance
Source: Signal Transduct Target Ther. 2025 Apr 21;10:127. doi: 10.1038/s41392-025-02210-1 (PMC12009988; doi:10.1038/s41392-025-02210-1)
Supplement: Supplementary file 1 — Supplementary File [file 41392_2025_2210_MOESM1_ESM.docx]

**Supplementary Materials for**

**Ku80K568 crotonylation conversion to SUMOylation facilitates DNA non-homologous end joining and radioresistance**

Hongling Zhao,^1#^ Shanshan Gao,^1,#^ Yang Han,^1^ Dafei Xie,^1^ Lihui Xuan,^1,2^ Xin Huang,^1^ Jinhua Luo,^1,2^ Qian Ran,^3^ Gang Li,^1,4^ Hejiang Guo,^1^ Weixiang Hu,^1^ Jin Jia,^1,4^ Xiaochang Liu,^1^ Yuhao Liu,^1^ Jinpeng Tan,^1,4^ Chenjun Bai,^1^ Yongqing Gu,^1^ Teng Ma,^5^ Zhongjun Li,^3^ Hua Guan,^1,*^ Ruixue Huang,^2,*^ Ping-Kun Zhou,^1,4,6,*^

^6^Lead contact: zhoupk@bmi.ac.cn (P.K.Z.)

*Correspondence: zhoupk@bmi.ac.cn (P.K.Z.), huangruixue@csu.edu.cn (R.H.), guanhua@bmi.ac.cn (H.G.)

**This PDF file includes:**

Parts of materials and methods

Supplementary Figures S1 to S23

Supplementary Table S1 to S2

Original and uncropped films of Western blots

**Parts of Materials and Methods**

KEY RESOURCES TABLE

| REAGENT or RESOURCE | SOURCE | IDENTIFIER |
| --- | --- | --- |
| Antibodies | | |
| Monoclonal ANTI-FLAG® M2 mouse antibody | Sigma‒Aldrich | Cat#F3165; RRID: AB_259529 |
| Crotonyllysine Mouse mAb | PTM Biolabs | Cat#PTM-502;  RRID:AB_2877695 |
| Anti-Acetyllysine Mouse mAb | PTM Biolabs | Cat# PTM-101; RRID: AB_2940830 |
| Anti-Ku80-K568cr | PTM Biolabs | N/A |
| β-actin Mouse mAb | ZSGB-Bio | Cat# TA-09;RRID: AB_2636897 |
| Anti-HA Tag Polyclonal Antibody | Abcam | Cat# ab9110; RRID:AB_307019 |
| Anti-GFP Tag Polyclonal antibody | Santa Cruz | Cat# sc-9996, RRID:AB_627695 |
| Anti-MYC Tag Polyclonal antibody | Proteintech | Cat#16286-1-AP; RRID: AB_11182162 |
| Anti-HDAC8 Rabbit pAb | Proteintech | Cat#17548-1-AP; RRID:AB_2116926 |
| Anti-DNA-PK Monoclonal Antibody (18-2) | Thermo scientific | Cat#MA5-13238; RRID: AB_10988612 |
| Anti-DNA PKcs (phospho-S2056) antibody | Abcam | Cat#ab18192; RRID: AB_869495 |
| Anti-phospho-Histone H2AX (Ser139) Antibody | Millipore | Cat#05-636; RRID: AB_309864 |
| Anti-Ku70 Antibody (E-5) | Santa Cruz | Cat#sc-17789; RRID: AB_628454 |
| Anti-Ku-86 Antibody (H-300) | Santa Cruz | Cat#sc-9034; RRID: AB_2218743 |
| Anti-PCAF Antibody (C-4) | Santa Cruz | Cat#sc-13124; RRID: AB_2128417 |
| Anti-SUMO2/3 Antibody | Proteintech | Cat# 11251-1-AP, RRID:AB_2198405 |
| Anti-CBX4 Antibody | Affinity Biosciences | Cat# DF13461, RRID:AB_2846480 |
|  |  |  |
| Anti-DNA-PK Antibody (G-4) | Santa Cruz | Cat#sc-5282, RRID:AB_2172848 |
| Anti-His-Tag(H-3) HRP Antibody | Santa Cruz | Cat# sc-8036, RRID:AB_627727 |
| GAPDH | Proteintech | Cat# 60004-1-lg; RRID: AB_2107436 |
| Crotonoyl coenzyme A trilithium salt | Sigma | Cat#28007 |
| Recombinant Human HDAC8 protein | Sigma | Cat#SRP0110 |
| Recombinant Human PCAF protein | Abcam | Cat#ab268695 |
| Recombinant Human Ku70 protein | This paper | N/A |
| Recombinant Human His-Ku80 WT protein | This paper | N/A |
| Recombinant Human His-Ku80 K568R protein | This paper | N/A |
| Recombinant Human Ku80 WT protein | This paper | N/A |
| Recombinant Human Ku80 K568R protein | This paper | N/A |
| H3 | Proteintech | Cat# 17168-1-AP; RRID: AB_2716755 |
| Alexa Fluor 488-labeled Goat Anti-Mouse IgG (H+L) | Invitrogen | Cat#A-11001; RRID: AB_2534069 |
| Alexa Fluor 568-labeled Goat Anti-Mouse IgG (H+L) | Invitrogen | Cat#A-11004; RRID:  AB_2534072 |
| Alexa Fluor 568-labeled Goat Anti-Rabbit IgG (H+L) | Invitrogen | Cat#A-11036; RRID: AB_10563566 |
| Chemicals, peptides, and recombinant proteins | | |
| Hydroxyurea | Sigma‒Aldrich | Cat#400046-5GM |
| Etoposide | Sigma‒Aldrich | Cat#E1383 |
| Camptothecin | Sigma‒Aldrich | Cat#C9911 |
| Mitomycin C | Sigma‒Aldrich | Cat#M0503 |
| Critical commercial assays | | |
| Annexin V-APC/7-AAD Apoptosis Detection Kit | Elabscience | Cat#E-CK-A218 |
| QuickMutation™ Plus Kit | Beyotime | Cat#D0208S |
| LightShift™ Chemiluminescent EMSA Kit | Thermo Fisher Scientific | Cat#20148 |
| Anti-Flag® M2 Affinity Gel | Sigma | Cat#AZ220 |
| HisSep Ni-NTA MagBeads | Yeasen Biotechnology | Cat# 20561ES08 |
| DAPI | ZSGB-BIO | Cat# ZLI-9557 |
| Lipofectamine 3000 | Thermo Fisher Scientific | Cat#L3000015 |
| Lipofectamine 2000 | Thermo Fisher Scientific | Cat#11668027 |
| PCI-34051 | Selleck | Cat#S2012 |
| PCI-34051 | MCE | Cat#HY-15224 |
| DNase I | Thermo Fisher Scientific | Cat#90083 |
| Hoechst 33342 | Thermo Fisher Scientific | Cat#62249 |
| Chromatin Extraction Kit | Abcam | Cat#ab117152 |
| TSA | Selleck | Cat#S1045 |
| NAM | Selleck | Cat#S1899 |
| DMSO | Sigma‒Aldrich | Cat#D2650 |
| NEM | Selleck | Cat#S3692 |
| Puromycin | Solarbio | Cat#P8230 |
| Blasticidin | Genomeditech | Cat#GM-040404 |
| G418 Sulfate | Genomeditech | Cat#GM-040402 |
| Protein A/G -agarose beads | Milipore | Cat# IP10 |
| Comet Assay Kit | R&D system a bioteche brand | Cat # 4250-050-K |
| NE-PER Nuclear and Cytoplasmic | Thermo Scientific | Cat # 78835 |
| Extraction Reagents |  |  |
| Bacterial and virus strains | | |
| DH5α | Tsingke Bio | Cat#TSC-C14 |
| BL21 | Tsingke Bio | Cat#TSC-E01 |
| Experimental models: Cell lines | | |
| HeLa | ATCC | Cat#CCL-2 |
| HEK-293T | ATCC | Cat#CRL-3216 |
| MCF-7 | ATCC | Cat#HTB-22 |
| A549 | ATCC | Cat#CRM-CCL-185 |
| HepG2 | ATCC | Cat#HTB-14 |
| U87 | ATCC | Cat#HB-8065 |
| shKu80 HeLa | This study | N/A |
| GFP-Flag-Ku80(WT) HeLa-Ku80 KO | This study | N/A |
| GFP-Flag-Ku80(K568R) HeLa-Ku80 KO | This study | N/A |
| Experimental models: Organisms/strains | | |
| BALB/c-nu mice | SPF (Beijing) Biotechnology | N/A |
| Oligonucleotides | | |
| Control siRNA: UUCUCCGAACGUGUCACGUTT | This paper | N/A |
| PCAF siRNA#1: GCGACAACUCCUGGAACAATT | This paper | N/A |
| PCAF siRNA #2: AAUCGCCGUGAAGAAAGCGCA | This paper | N/A |
| HDAC8 siRNA #1: GGUGUACAUAGCCUUUAAUTT | This paper | N/A |
| HDAC8 siRNA #2: GACGGAAAUUUGAGCGUAUUCUCU | This paper | N/A |
| CBX4 siRNA: TGAAGATAGTCAAGAACAATT | This paper | N/A |
| Recombinant DNA | | |
| Plasmid: pcDNA-3.1-Myc-SIRT1 | This paper | N/A |
| Plasmid: pcDNA-3.1-Myc- SIRT2 | This paper | N/A |
| Plasmid: pcDNA-3.1-Myc- SIRT3 | This paper | N/A |
| Plasmid: pcDNA-3.1-Myc-HDAC1 | This paper | N/A |
| Plasmid: pcDNA-3.1-Myc-HDAC3 | This paper | N/A |
| Plasmid: pcDNA-3.1-Myc-HDAC8 | This paper | N/A |
| Plasmid: His-Ku80 WT | This paper | N/A |
| Plasmid: His-Ku80 K568R | This paper | N/A |
| Plasmid: pcDNA-3.1-Flag-Ku80 | This paper | N/A |
| Plasmid: pcDNA-3.1-Flag-Ku80 K568R | This paper | N/A |
| Plasmid: pcDNA-3.1- EGFP-Flag-Ku80 | This paper | N/A |
| Plasmid: pcDNA-3.1-EGFP-Flag-Ku80 K568R | This paper | N/A |
| Plasmid: Myc-GCN5 | This paper | N/A |
| Plasmid: Flag-PCAF, Flag -TIP60 | Gifted from Jiadong Wang’s laboratory | N/A |
| Plasmid: HA-CBP, HA-P300 | This paper | N/A |
| Plasmid: Myc-PIAS1, Myc-PIAS4, Myc-CBX4 | Gifted from Jiadong Wang’s laboratory | N/A |
| Plasmid: HA-PIAS3 | Gifted from Xiaofeng Zheng’s laboratory | N/A |
| Plasmid: Flag-Ku80 S577A | This paper | N/A |
| Plasmid: Flag-Ku80 S580A | This paper | N/A |
| Software and algorithms | | |
| GraphPad Prism v8.0 | GraphPad Software | https://www.graphpad.com/scientific-software/prism/ |
| ImageJ | NIH | https://imagej.nih.gov/ij |
| Adobe Illustrator 2020 | Adobe | https://www.adobe.com/ |
| Adobe Photoshop 2020 | Adobe | https://www.adobe.com/ |
| NIS ELEMENTS | Nikon | https://www.microscope.healthcare.nikon.com/ |
| Micro-Manager | Edelstein, et al. | https://micro-manager.org/ |

**Co-IP, western blotting and nucleoplasmic isolation experiment**

For the co-IP assay, cells were harvested and then lysed in NETN buffer (300 mM NaCl, 20 mM Tris-base, 1 mM EDTA, and 0.5% (v/v) NP-40) supplemented with protease inhibitor cocktail (cOmplete). After 20 min of constant agitation at 4 °C, the cell lysates were subjected to 10 min of centrifugation at 12,000 rpm, harvested, and the supernatant was incubated with the corresponding antibodies or beads for 6 h with rotation at 4 °C. Thereafter, the samples were washed three times in NETN buffer supplemented with protease inhibitor. The final immunoprecipitant samples were separated by SDS–polyacrylamide gel electrophoresis (SDS–PAGE) and detected with the indicated antibodies. For the SUMOylation assay Co-IP, cells were harvested and then lysed in NETN buffersupplemented with fresh protease inhibitor cocktail (cOmplete) and 20 mM NEM (Selleck). For Western blotting, cells were harvested and then lysed in NETN buffer supplemented with protease inhibitor cocktail (cOmplete) and phosphatase inhibitors (cOmplete). After 20 min of constant agitation at 4 °C, the cell lysates were subjected to 10 min of centrifugation at 12,000 rpm. The final samples were separated by SDS-PAGE and detected with the indicated antibodies. Three independent replications of WB experiments were carried out. For the nucleoplasmic isolation experiment, the cells were sampled and the corresponding lysates were added according to the instructions of the cytoplasmic nuclear extraction kit (Thermo Scientific) to extract the cytoplasm and nucleus, which were further subjected to western blotting and IP.

**Crotonylation modification omics assay**

MCF-7 cells were divided into two groups, control group (MCF-7 NR) and radiation (8Gy) treatment group (MCF-7 IR) (Fig.1a). Cells were harvested at 4h after radiation and crotonylation modification omics were performed by PTM BIO Ltd. Company, Shanghai, China. Three replicates of each group were analyzed. The steps include protein extraction, trypsin hydrolysis, modification enrichment, liquid chromatography-mass spectrometry analysis (LC-MS/MS).

For modification enrichment, we dissolved the peptide segments in IP buffer solution (100 mM NaCl, 1 mM EDTA, 50 mM Tris HCl, 0.5% NP-40, pH 8.0), transferred the supernatant to pre washed crotonylation resin (antibody resin item number PTM-503, sourced from Hangzhou Jingjie Biotechnology Co., Ltd., PTM Bio), placed on a rotating shaker at 4 ℃, gently shake and incubate overnight. After incubation, washed the resin four times with IP buffer solution and twice with deionized water. Finally, use 0.1% trifluoroacetic acid eluent to elute the peptide segments bound to the resin, three times in total. Collectted the eluent and vacuum freeze dry it. After draining, desalted according to the C18 ZipTips instructions, vacuum freeze and dry, and provide for liquid chromatography-mass spectrometry analysis.

For liquid chromatography-mass spectrometry analysis, the peptide segments were dissolved in liquid chromatography mobile phase A and separated using the NanoElute ultra-high performance liquid chromatography system. Mobile phase A was an aqueous solution containing 0.1% formic acid and 2% acetonitrile; Mobile phase B was a solution containing 0.1% formic acid and 100% acetonitrile. Liquid phase gradient setting: 0-42 min, 6%~24% B; 42-54 min, 24%~33% B; 54-57 min, 33%~80%B, 57-60 min, 80% B, maintained at a flow rate of 300nL/min. The peptide segments were separated by an ultra-high performance liquid chromatography system and injected into a Capillary ion source for ionization before being analyzed by timsTOF Pro mass spectrometry. The ion source voltage was set to 1.8 kV, and the peptide parent ions and their secondary fragments were detected and analyzed using high-resolution TOF. The scanning range of secondary mass spectrometry was set to 400-1500 m/z. The data collection mode used Parallel Cumulative Serial Fragmentation (PASEF) mode. After collecting a primary mass spectrum, 10 PASEF mode were used to collect secondary spectra of the parent ion charge within the range of 0-5. The dynamic exclusion time for tandem mass spectrometry scanning was set to 30 seconds to avoid repeated scanning of the parent ion.

The result of MS/MS data were processed using MaxQuant with an integrated Andromeda search engine (v.1.5.2.8). Tandem mass spectra were searched against the human Swiss-Prot database (20,203 sequences) concatenated with the reverse decoy database. Trypsin/P was specified as a cleavage enzyme, allowing up to four missing cleavages. The mass tolerance for precursor ions was set to 20 parts per million in the first search, and 5 parts per million in the main search, and the mass tolerance for fragment ions was set to 0.02 Da. Carbamidomethyl on Cys was specified as a fixed modification, and Kcr modification and oxidation on Met were specified as variable modifications. The false discovery rate (FDR) was adjusted to <1% and the minimum score for modified peptides was set to >40.

**In vitro crotonylation and decrotonylation assay**

In vitro crotonylation assays were performed with 1 µg Ku80 WT, 0.2 µg PCAF, and 300 µM crotonyl-CoA mixed with reaction buffer (50 mM Tris-Cl, pH 7.5, 100 mM NaCl, 1 mM EDTA, 1 mM DTT) in a final volume of 50 µl. Reactions were performed at 37 °C for 1 h. Then, the enzymes were inactivated at 70 °C for 5 min. 0.2 µg of HDAC8 was added to the samples and incubated at 37 °C for decrotonylation. The samples were separated by SDS‒PAGE and detected by Western blotting using an anti-Kcr antibody.

**Electrophoretic mobility shift assay (EMSA)**

The Ku80 WT, Ku80 K568R, and Ku70 proteins were expressed and purified from BL21 *E. coli* by Zoonbio Biotechnology, Ltd. Company, Nanjing, China. Then, EMSAs were performed by using an electrophoretic mobility shift assay (EMSA) kit (Thermo Fisher #20148) according to the manufacturer’s protocol. Briefly, the indicated concentrations of proteins were mixed with biotin-labeled DNA (2 fM) substrate in 20 μL of reaction mixture (2.5% glycerol, 5 mM MgCl_2_, 50 ng/μL poly (dI.dC), 0.05% NP-40). Then, the samples were incubated at room temperature for 20 mins. Finally, the samples were separated by 6% native polyacrylamide gels in 0.5× TBE buffer (2 mM EDTA, 90 mM Tris-HCl, 90 mM boric acid, pH 8.3) and detected by an HRP-conjugated anti-biotin antibody using a GE ImageQuant™ LAS 500 Imaging System.

**His-pull down assay**

For the Ku80 WT/K568R His pull-down assays, the fusion proteins were expressed and purified in BL21 E. coli using Ni-IDA Sepharose 6FF (His-Tag) produced by Zoonbio Biotechnology, Ltd. Company, Nanjing, China. Then, 4 µg of purified fusion protein was incubated with cell lysates at 4 °C for 2 hours, mixed with 20 µl of His beads, and incubated at 4 °C for 3 hours. The samples were separated by SDS‒PAGE and analyzed by western blotting.

**NHEJ DNA repair assay**

NHEJ assay was used to determine the efficiency of NHEJ repair. Briefly, shKu80 HeLa cells integrated with direct repeat GFP (DR-GFP) were infected with the indicated plasmid. Then, the cells were transfected with the I-Scel and p-cherry expression vectors. Doxycycline (DOX) was added to induce I-SecI expression. Forty-eight hours after DOX was added, the percentage of GFP- or RFP-positive cells was analyzed by fluorescence-activated cell sorting (FACS). NHEJ efficiency was presented by GFP-positive cells as percentages of RFP- positive cells, respectively. Repair frequencies are presented as the means ± SDs of at least three independent experiments.

**Immunofluorescence assay**

GFP-Flag-Ku80 (WT) and GFP-Flag-Ku80 (K568R) HeLa-Ku80 KO cells and shKu80 cells were cultured on coverslips and treated with 8 Gy of IR. MCF-7, HeLa, HepG2, A549, and U87 cells were cultured on coverslips and treated with HDAC8 inhibitor PCI-34051 for 6 hours and then treated with 8 Gy of IR. After IR, the cells were washed three times with ice-cold PBS at different time points and then incubated with 4% paraformaldehyde at room temperature for 15 min. The cells were subsequently permeabilized with PBS containing 0.5% Triton X-100 at room temperature for 10 min and blocked with 3% BSA in PBS at room temperature for 1 hour. Then, the cells were incubated for 1 hour with primary antibodies at room temperature. The cells were subsequently washed three times with PBS and then incubated with secondary antibodies. Then, the slides were subjected to three washes in PBS and 10 min of DAPI staining at room temperature for nuclear DNA visualization.

Finally, coverslips were placed on anti-fade buffer-containing slides, and a fluorescence microscope (Nikon) was used for visualization of the results. More than 50 cells per sample were analyzed at each independent experiment, and representative images are shown.

**Laser micro-irradiation assay**

GFP-Flag-Ku80 (WT) HeLa-Ku80 KO cells transected with PCAF siRNA or treated with PCI-34051 and GFP-Flag-Ku80 (K568R) HeLa-Ku80 KO cells were plated into 35-mm glass bottom dishes (MatTek Corporation). Laser micro-irradiation (UV laser with a wavelength of 365nm) was performed on a NIKON Ti2 microscope equipped with Ilas Pulse (GATACA SYSTEMS). The laser output was set to 7.5%, which can reproducibly give focused GFP stripes. Images were taken every minute for 5 min following damage by using Micro Manager software. Quantitation of GFP localization (signal of GFP at bands of micro-irradiation) was performed using Image J software. For each condition, 20 biological replicates were performed.

**Clony forming assay**

For colony formation, GFP-Flag-Ku80 (WT) and GFP-Flag-Ku80 (K568R) HeLa-Ku80 KO cells were seeded in six-well plates and treated with the indicated irradiation at predesigned doses. Digested the cells of each treatment group with trypsin and perform cell counting; Diluted the cells to an appropriate concentration, and spread 1500 cells into a 6-well plate. On the second day after laying, drugs (HU; Sigma‒Aldrich #400046-5GM; 1 mM), etoposide (Sigma‒Aldrich #E1383; 100 nM), campathecin (Sigma‒Aldrich #C9911; 1 μM) or mitomycin C (Sigma‒Aldrich #M0503; 5 μM)) treatment were carried out, and all cells were immediately returned to the incubator for further cultivation after treatment. After 2 weeks of cultivation, remove the cells (or when cell clone formation is clearly observed), discard the supernatant of the culture medium, wash three times with PBS, and discard PBS. Add 2 mL of 4% paraformaldehyde to fix the cells, discard the supernatant, and wash three times with PBS. Add 2 mL of crystal violet staining, discard the supernatant, and wash three times with PBS. Shake the liquid in the culture well dry and invert the culture plate to air dry. Scan the culture plate with a scanner, save the images, and perform statistical analysis on the formation of clones in each group.

**Proliferation and cell viability assays**

The proliferation ability of different cancer cells was monitored by using the xCELLigence Real-Time Cell Analyzer (RTCA)-MP system (Acea Biosciences/Roche Applied Science). This platform is able to measure cellular growth status in real-time. Then, 50 µl of culture medium was added to each well of an E-Plate 96 (Roche Applied Science) to reach equilibrium. GFP-Flag-Ku80 (WT) and GFP-Flag-Ku80 (K568R) HeLa-Ku80 KO cells, MCF-7, HeLa, HepG2, A549, and U87 cells were incubated in 60 mm culture plates for 24 h, after which 3,000 cells in 100 µl of culture medium were seeded in an E-Plate 96. MCF-7, HeLa, HepG2, A549, and U87 cells were cultured for 12 h and then treated with HDAC8 inhibitor PCI-34051(10μm) for 6 h, followed by 8 Gy of IR. GFP-Flag-Ku80 (WT), GFP-Flag-Ku80 (K568R) HeLa-Ku80 KO cells were directly treated with 8 Gy ionizing radiation after 12h of culture. The E-Plate 96 was locked in a Real-Time Cell Analysis (RTCA)-multiple plates (MP) device at 37 °C with 5% CO_2_. Changes in electrical impedance were measured as a cell index that directly reflects cellular proliferation on biocompatible microelectrode-coated surfaces. The cell index was read automatically every 15 min, and the recorded curve is shown as the cell index ± SEM. For the cell viability assay, 3,000 cells were seeded in an E-Plate 96 and treated with HU (Sigma‒Aldrich #400046-5GM; 1 mM), etoposide (Sigma‒Aldrich #E1383; 100 nM), campathecin (Sigma‒Aldrich #C9911; 1 μM) or mitomycin C (Sigma‒Aldrich #M0503; 5 μM).

**Flow cytometry**

The GFP-Flag-Ku80 (WT) and GFP-Flag-Ku80 (K568R) HeLa-Ku80 KO cells were trypsinized and washed with ice-cold PBS. For analysis of apoptosis, the Annexin V-APC/7-AAD Apoptosis Detection Kit (Cat# E-CK-A218) was used according to the manufacturer’s instructions. FACS analysis was performed by flow cytometry, and the percentage of apoptotic cells was recorded using a FACSCalibur flow cytometer (ACEA Biosciences).

**Xenograft experiment and PDX (Patient-derived tumor xenograft) models**

Four-week-old female BALB/c-nu mice were purchased from SPF Biotechnology Co., Ltd. (Beijing) and housed in a specific pathogen-free animal facility at the Experimental Animal Center of the Academy of Military Medical Sciences with autoclaved food, bedding, and water. Mice were housed 5-6 per cage in a temperature- and humidity-controlled room. The animal experiments were performed in accordance with the Laboratory Animal Guidelines of Welfare and Ethics.

For the xenograft experiment, GFP-Flag-Ku80 (WT) and GFP-Flag-Ku80 (K568R) HeLa-Ku80 KO cells were generated and collected, and the cell concentration was adjusted to 1 × 10^7^ cells/ml with PBS. Then, 100 μl of cell solution was subcutaneously injected into the right oxter of female nude mice (five mice in each group). Three days later, the mice were locally irradiated with 8 Gy γ-rays or left unirradiated. We observed tumor growth every 3 days, and the tumor volume was calculated using the following formula: tumor volume = length × width^2^/2. Approximately 3 weeks after injection, the tumors were removed, and the weights of the tumors were calculated. Animal experiment of xenograft was approved by the Animal Care and Use Committee of the Academy of Military Medical Sciences (IACUC-DWZX-2024-529).

For PDX experiment, lung cancer or meningioma patients-derived xenografts (PDXs) were cut into 1 mm^3^, and 2-3 xenografts were subcutaneously transplanted into 5-week-old male BALB/c-nude mice (SPF Biotechnology Co., Ltd.). One week after subcutaneous transplantation, mice were randomized into 4 groups: vehicle control group (saline), vehicle + IR group (8 Gy), PCI-34051 group (0.5 mg/kg), and PCI-34051 (0.5 mg/kg) + IR (8 Gy) group. PCI-34051 or saline was administrated intraperitoneally before irradiation. Tumor volume was measured every 3 days following the formula: tumor volume=length×width^2^/2. Approximately 3 weeks after irradiation, the mice were euthanized, and the tumor was collected for subsequent research.

**Supplemental Figures legends:**

**Supplementary Figure 1. Quantitative crotonylome profiling of lysine crotonylated proteins in MCF-7 cells responding to ionizing radiation**

**a** Motif enrichment heatmap showing the frequency of amino acids around the identified Kcr sites using the MoMo analysis tool based on the motif-x algorithm. **b** Pie chart showing the distribution of the number of identified Kcr sites per protein. **c** IceLogo is a tool used to analyze and visualize patterns and preferences within sequence data, which shows preferences for flanking sequences at Kcr sites as indicated. **d** Venn diagram comparing the total numbers of Kcr sites identified in previous studies and in our study. **e** Scatterplot showing the quantification of Kcr sites in relation to peptide intensities compared between irradiated and nonirradiated MCF-7 cells. **f** Clusters of Orthologous Groups (COG)/GO functional classification analysis of differentially expressed Kcr proteins. **g** LC‒MS/MS spectrum of XRCC5/Ku80 peptides containing crotonylated lysine 338.

**Supplementary Figure 2. Screening of Ku80-KO cells stably expressing exogenous GFP-Flag-tagged WT Ku80 or K568 mutant and confirming of the specific anti-Ku80 K568cr antibody**

**a**	Changes of Ku80 crotonylation levels in HeLa cells at 1 to 4 h post-irradiation of 8 Gy γ-ray, analyzed by IP coupling western blotting analysis. **b** Flow cytometry screening of stable cell lines in Ku80-knockout HeLa cells harboring exogenous GFP-Flag-tagged wild-type Ku80 (WT) or mutant Ku80 (K568R). **c** The specificity of the antibody against Ku80 K568cr was verified by dot blot assays. **d** Downregulation of Ku80 K568 Kcr was confirmed by anti-Ku80-K568cr antibody analysis in cells irradiated with 8 Gy γ-rays. Three independent replications of WB experiments. β-actin was assayed to ensure equivalent loading and transfer.

**Supplementary Figure 3. Identification of PCAF as a mediator for Ku80 crotonylation**

**a** Identification of crotonylase mediating Ku80 crotonylation. A series of crotonylase candidates (acetyltransferases) expressing vectors were transfected with Flag-Ku80 vectors into HEK293T cells, respectively, the crotonylation level of Ku80 was analyzed by IP and western blotting. **b** The interaction of Ku80 and PCAF after irradiation was analyzed by IP and western blotting. Three independent replications of WB experiments. β-actin was assayed to ensure equivalent loading and transfer.

**Supplementary Figure 4. Analysis of three-dimensional structures for the interaction of Ku80 K568 /K568cr /K568R with DNA-PKcs, Ku70, and dsDNA**

**a-c** Laser micro-irradiation assays of Ku80 recruitments to DNA damage sites. **a** Stable cell lines in Ku80-knockout HeLa cells harboring exogenous GFP-Flag-tagged wild-type Ku80 (WT) or mutant Ku80 (K568R); **b** HDAC8-specific inhibitor PCI-34051 (10 μM, 6 h)-treated GFP-Flag-Ku80 (WT) HeLa-Ku80 KO cells; **c** GFP-Flag-Ku80 (WT) HeLa-Ku80 KO cells with PCAF knockdown by siRNA were subjected to laser micro-irradiation. The GFP stripes were quantitated. The average of 10 replicates were measured and analyzed. Error bars represent standard deviation: NS indicates *P* ≥ 0.05. **d-e** Recruitment of Ku80 to chromatin in HeLa cells after 10 μM PCI-34051 treatment (**d**) or PCAF knockdown by siRNA (**e**) was analyzed. **f** The three-dimensional structure of crotonylated and mutated Ku80 interacting with Ku70 compared to that of the original Ku80 at the K568 site. **g** The three-dimensional structure of crotonylated and mutated Ku80 interacting with Ku70 and DNA-Kcs compared to that of the original Ku80 at the K568 site. **h** The three-dimensional structure of crotonylated and mutated Ku80 interacting with Ku70 and dsDNA compared to that of the original Ku80 at the K568 site. **i** Western blotting analysis of DNA-PKcs Thr2609 phosphorylation in stable cell lines of Ku80-knockout HeLa cells expressing exogenous Ku80 (WT) or mutant Ku80 (K568R) at 1 to 4h post-irradiation with 8Gy γ-ray. Three independent replications of WB experiments. β-actin was assayed to ensure equivalent loading and transfer.

**Supplementary Figure 5. Identification of CBX4 is an enzyme to mediate SUMOylation of Ku80**

**a** Ku80-knockout HeLa cells harboring exogenous GFP-Flag-tagged wild-type Ku80 (WT) were transferred with Myc-CBX4, and western blotting was performed on the CoIP products of Flag tag antibody using Myc tag antibody. **b** CoIP assay of the interaction between endogenous Ku80 and CBX4 in HeLa cells. The CoIP products of the CBX4 antibody were subjected to western blotting analysis of Ku80 in HeLa cells. αCBX4 represents CBX4 antibody. **c** Confirmation of CBX4 and PCAF depression by the corresponding specific siRNAs, analyzed by western blotting. Three independent replications of WB experiments. β-actin was assayed to ensure equivalent loading and transfer.

**Supplementary Figure 6. Mutation of Ku80 K568R results in defective repair of DNA DSBs induced by irradiation as indicated by increased residual γH2X level**

Ku80-KO HeLa cells were transfected with GFP-Flag-Ku80 WT and Flag-Ku80 K568 mutant expressing vectors, respectively. Western blotting analysis was performed on the dynamic changes of γH2X levels in the cells at 1 to 12h post-irradiation with 8Gy γ-ray. Three independent replications of WB experiments. β-actin was assayed to ensure equivalent loading and transfer.

**Supplementary Figure 7. Ku80 K568 crotonylation and SUMOylation were assessed with or without radiation treatment in multiple cancer cell lines.** **a** Representative gel and blots were shown in A549 cancer cells, three dependent replications of experiment. **b** Quantitative analysis of (**a**). **c** Representative gel and blots were shown in HepG2 cancer cells, three dependent replications of experiment. **d** Quantitative analysis of (**c**). **e** Representative gel and blots were shown in U87 cancer cells, three dependent replications of experiment. **f** Quantitative analysis of (**e**). Date are presented as mean±SD. NC: non Radiation. **p* < 0.05, ***p* < 0.01. β-actin was assayed to ensure equivalent loading and transfer.

**Supplementary Figure 8. Ku80 K568 crotonylation and SUMOylation were assessed in multiple cancer cell lines treatment with IR and PCI-34051.** **a** Representative gel and blots were shown in A549 cancer cells, three dependent replications of experiment. **b** Quantitative analysis of (**a**). **c** Representative gel and blots were shown in HepG2 cancer cells, three dependent replications of experiment. **d** Quantitative analysis of (**c**). **e** Representative gel and blots were shown in U87 cancer cells, three dependent replications of experiment. **f** Quantitative analysis of (**e**). Data are represented as mean±SD. NC: non Radiation. ***p* < 0.01, ****p* < 0.001. ns= non-significant difference. β-actin was assayed to ensure equivalent loading and transfer.

**Supplementary Figure 9. Effect of HDAC8-specific inhibitor PCI-34051 on γH2AX foci in irradiated A549 cells.** **a** The cells were treated with HDAC8-specific inhibitor PCI-34051 (10 μM, 6 h) or DMSO solvent as control, and γH2AX foci were observed at indicated time-points by IF assay after IR. **b** Quantification of γH2AX foci numbers affected PCI-34051. 50 cells were quantified and analyzed. NR: non Radiation. Data are represented as mean±SD. **p* < 0.05, ***p* < 0.01.

**Supplementary Figure 10. Effect of HDAC8-specific inhibitor PCI-34051 on** γ-H2AX **foci formation in irradiated HepG2 cells.** **a** The cells were treated with HDAC8-specific inhibitor PCI-34051 (10 μM, 6 h) or DMSO solvent as control, and γ-H2AX foci were observed at indicated time-points by IF assay after IR. **b** Quantification of γH2AX foci formation affected PCI-34051. 50 cells were quantified and analyzed.NR: non Radiation. Data are represented as mean±SD. **p* < 0.05, ***p* < 0.01.

**Supplementary Figure 11.** **Effect of HDAC8-specific inhibitor PCI-34051 on** γH2AX **foci formation in irradiated U87 cells.** **a** The cells were treated with HDAC8-specific inhibitor PCI-34051 (10 μM, 6 h) or DMSO solvent as control, and γH2AX foci were observed at indicated time-points by IF assay after IR. **b** Quantification of γH2AX foci formation affected PCI-34051. 50 cells were quantified and analyzed. NR: non Radiation. Data are represented as mean±SD. **p* < 0.05.

**Supplementary Figure 12. Effect of HDAC8-specific inhibitor PCI-34051 on** γH2AX **foci formation in irradiated HeLa cells. a** The cells were treated with HDAC8-specific inhibitor PCI-34051 (10 μM, 6 h) or DMSO solvent as control, and γH2AX foci were observed at indicated time-points by IF assay after IR. **b** Quantification of γH2AX foci formation affected PCI-34051. 50 cells were quantified and analyzed. NR: non Radiation. Data are represented as mean±SD. **p* < 0.05, ***p* < 0.01.

**Supplementary Figure 13. Effect of HDAC8-specific inhibitor PCI-34051 on** γH2AX **foci formation in irradiated MCF-7 cells.** **a** The cells were treated with HDAC8-specific inhibitor PCI-34051 (10 μM, 6 h) or DMSO solvent as control, and γH2AX foci were observed at indicated time-points by IF assay after IR. **b** Quantification of γH2AX foci formation affected PCI-34051. 50 cells were quantified and analyzed. Data are represented as mean±SD. **p* < 0.05.

**Supplementary Figure 14. EMSAs were performed with the dsDNA, Ku80 WT, or K568R and Ku70 proteins.** **a-c** Three independent replications of experiment. **d** Relative expression of protein-DNA complex for the indicated groups was quantified and analyzed. Data presented as Mean ±SD. **p* < 0.05. **e** In [eukaryotic cells](https://www.sciencedirect.com/topics/immunology-and-microbiology/eukaryotic-cell), as Choon Kit Tang et al wrote in the book of *Biological DNA Sensor*,2014, both [Ku proteins](https://www.sciencedirect.com/topics/biochemistry-genetics-and-molecular-biology/ku-protein) consist of three domains: an [N-terminal](https://www.sciencedirect.com/topics/biochemistry-genetics-and-molecular-biology/n-terminus) von Willebrand A domain (vWA), a central [DNA binding](https://www.sciencedirect.com/topics/biochemistry-genetics-and-molecular-biology/dna-binding) domain, and a diverged [C-terminal](https://www.sciencedirect.com/topics/biochemistry-genetics-and-molecular-biology/c-terminus) domain (CTD). Data are represented as mean±SD. **p* < 0.05.

**Supplementary Figure 15. NHEJ efficiency was determined using the EJ5-GFP reporter assay in shku80 cells transfected with Flag-tagged WT Ku80 or the point mutant Ku80-K568R.** The data are presented as the means ± SDs from three independent experiments. Data presented as Mean ±SD, two-tailed Student’s t-test. A *p*-value less than 0.05 indicates a significant difference.

**Supplementary Figure 16. Neutral comet assay**. **a** Representative images of Neutral comet assay. Scale bar, 10 μm. **b** Quantitative analysis of neutral comet results. **c** Quantitative analysis of relative tail moment results (considering the account the difference in the initial moment). Data presented as Mean ±SD.** *p* < 0.01.

**Supplementary Figure 17.** Quantitative analysis of the sensitivity of Ku80 WT and Ku80 K568R mutant HeLa cells to DNA damage or replication stress-inducing agents at 72h was determined by the xCELLigence Real-Time Cell Analyzer (RTCA)-MP system. **a** ETO vs. DMSO. **b** CPT vs. DMSO. **c** HU vs. DMSO. **d** MMC vs. DMSO. ETO: etoposide; CPT: camptothecin; HU: hydroxyurea; MMC: mitomycin C. Data presented as Mean ±SD. A *p*-value less than 0.05 indicates a significant difference. Three times replications.

**Supplementary Figure 18.** **Quantitative analysis of tumor doubling time was detected among indicated treatment groups.** n=5 each group. Data presented as Mean ±SD. A *p*-value less than 0.05 indicates a significant difference.

**Supplementary Figure 19.** Ku80 K568cr and SUMOylation within cytoplasm and nucleus, respectively in HeLa cells treated with or without radiation was detected by nucleoplasmic isolation experiment and further subjected to western blotting and IP.

**Supplementary Figure 20. The phosphorylation at S577 and S580 within Ku80 near Lys568 does not affect crotonylation and subsequent SUMOlysation.** Flag-Ku80 S577A and Flag-Ku80 S580A were transferred into HeLa cells. The cells were harvested at 1 h post 8Gy radiation. **a-c** Representative images of gels by Western blotting. **d** Quantitative analysis of Western blotting results. Data presented as Mean ±SD. * *p* < 0.01, ns: not significant. Three independent replications of WB experiments. β-actin was assayed to ensure equivalent loading and transfer.

**Supplementary Figure 21. PDX models. a** Lung cancer patient-derived xenografts were transplanted into 5-week-old male BALB/c-nude mice. mice were randomized into 4 groups: vehicle control group (saline), vehicle + IR group (8 Gy), PCI-34051 group (0.5 mg/kg), and PCI-34051 (0.5 mg/kg) + IR (8 Gy) group. n=5. **b** cancer tissues’ photo. **c** Tumor weight assay. **d** Tumor volume assay. **e** Meningioma cancer patient-derived xenografts were transplanted into 5-week-old male BALB/c-nude mice. mice were randomized into 4 groups: vehicle control group (saline), vehicle + IR group (8 Gy), PCI-34051 group (0.5 mg/kg), and PCI-34051 (0.5 mg/kg) + IR (8 Gy) group. n=3. F. cancer tissues’ photo. **g** Tumor weight assay. **h**. Tumor volume assay. n=5 each group. Data presented as Mean ±SD. A *p*-value less than 0.05 indicates a significant difference.

**Supplementary Figure 22.** IP analysis of the changes in Ku80 crotonylation 5 to 60 min after-8 Gy γ irradiation in HeLa-Ku80 KO cells expressing GFP-Flag-tagged Ku80 constructs.

**Supplementary Figure 23.** Clony forming assay for Fig 7e. **a** Representative images of cloning formation assay within Ku80WT and Ku80 K568R treated with DMSO, CPT, HU, ETO and MMC, respectively. **b** Quantitative assay of formation rate. **c** Quantitative assay of survival fraction. ***p*<0.01.

**Table S1 Crotonylated sites in DNA DSB repair proteins identified by**

**LC-MS/MS**

| **Table S1** Crotonylated sties in DNA DSB repair proteins identified by LC-MS/MS | | | | | | |
| --- | --- | --- | --- | --- | --- | --- |
| Modification sites information | | | | |  | |
| Gene name | Position | PEP | Score | Modified sequence | IR/ Ctrl Ratio | IR/ Ctrl P value |
| XRCC6 | 100K | 7.31E-37 | 236.67 | NSVNFK(1)NIYVLQELDNPGAK | 1.317 | 0.057111 |
| XRCC6 | 207K | 0.00106 | 76.255 | K(1)PGGFDISLFYR | 1.262 | 0.248671 |
| XRCC6 | 556K | 2.21E-05 | 160.62 | RPK(1)VEYSEEELK | 1.211 | 0.004065 |
| XRCC6 | 461K | 4.78E-05 | 224.68 | IMATPEQVGK(1)MK | 1.158 | 0.048261 |
| XRCC6 | 392K | 0.000527 | 108.4 | CLEK(1)EVAALCR | 1.136 | 0.355273 |
| XRCC6 | 565K | 5.62E-08 | 132.9 | VEYSEEELK(1)THISK | 1.126 | 0.161576 |
| XRCC6 | 94K | 0.004821 | 99.305 | DK(1)NSVNFK | 1.101 | 0.159929 |
| XRCC6 | 206K | 2.98E-19 | 203.8 | DTGIFLDLMHLK(1)K | 1.079 | 0.552529 |
| XRCC6 | 189K | 0.016041 | 101.41 | TK(1)AGDLR | 1.035 | 0.632781 |
| XRCC6 | 123K | 7.38E-63 | 295.37 | ILELDQFK(1)GQQGQK | 1.014 | 0.934367 |
| XRCC6 | 331K | 4.53E-06 | 171.09 | QIILEK(1)EETEELK | 1.007 | 0.986015 |
| XRCC6 | 338K | 0.000223 | 159.46 | EETEELK(1)R | 0.986 | 0.833254 |
| XRCC6 | 114K | 2.13E-07 | 135.21 | NIYVLQELDNPGAK(1)R | 0.981 | 0.844388 |
| XRCC6 | 238K | 5.54E-09 | 216.53 | VHFEESSK(1)LEDLLR | 0.946 | 0.497059 |
| XRCC6 | 570K | 0.000147 | 187.1 | THISK(1)GTLGK | 0.934 | 0.347089 |
| XRCC6 | 182K | 3.17E-15 | 112.68 | IMLFTNEDNPHGNDSAK(1)ASR | 0.929 | 0.322725 |
| XRCC6 | 317K | 5.82E-31 | 223.26 | TFNTSTGGLLLPSDTK(1)R | 0.925 | 0.628307 |
| XRCC6 | 463K | 0.024426 | 93.561 | MK(1)AIVEK | 0.861 | 0.006712 |
| XRCC6 | 287K | 0.003861 | 67.507 | ALKPPPIK(1)LYR | 0.81 | 0.141442 |
| XRCC6 | 605K | 1E-08 | 225.89 | KQELLEALTK(1)HFQD | 0.738 | 0.014009 |
| XRCC6 | 539K | 0.000512 | 73.891 | ELVYPPDYNPEGK(1)VTK | 0.666 | 0.137293 |
| XRCC5 | 282K | 0.000736 | 155.57 | TWTVVDAK(1)TLK | 1.554 | 0.09123 |
| XRCC5 | 155K | 0.000176 | 119.94 | SQLDIIIHSLK(1)K | 1.4 | 0.147218 |
| XRCC5 | 195K | 3.93E-05 | 56.08 | LGGHGPSFPLK(1)GITEQQK | 1.299 | 0.152218 |
| XRCC5 | 543K | 0.000262 | 154.81 | TLFPLIEAK(1)K | 1.179 | 0.083601 |
| XRCC5 | 202K | 0.00025 | 105.08 | GITEQQK(1)EGLEIVK | 1.175 | 0.135674 |
| XRCC5 | 702K | 3.57E-33 | 248.59 | EEASGSSVTAEEAK(1)K | 1.142 | 0.209329 |
| XRCC5 | 144K | 3.04E-16 | 211.52 | FSK(1)SQLDIIIHSLK | 1.125 | 0.271687 |
| XRCC5 | 665K | 0.000482 | 104.39 | ALQEK(1)VEIK | 1.123 | 0.276857 |
| XRCC5 | 332K | 0.000433 | 142.04 | VDEEQMK(1)YK | 1.059 | 0.310529 |
| XRCC5 | 648K | 0.000269 | 153.33 | EEAIK(1)FSEEQR | 1.023 | 0.768649 |
| XRCC5 | 565K | 1.52E-24 | 145.73 | DQVTAQEIFQDNHEDGPTAK(1)K | 0.966 | 0.80417 |
| XRCC5 | 545K | 1.19E-49 | 171.03 | K(0.093)K(0.907)DQVTAQEIFQDNHEDGPTAK | 0.932 | 0.597071 |
| XRCC5 | 534K | 0.000281 | 172.1 | IK(1)TLFPLIEAK | 0.896 | 0.344973 |
| XRCC5 | 660K | 2.03E-26 | 234.28 | FNNFLK(1)ALQEK | 0.822 | 0.095095 |
| XRCC5 | 532K | 0.002364 | 109.88 | SQIPLSK(1)IK | 0.742 | 0.021155 |
| XRCC5 | 568K | 3.11E-29 | 75.669 | LK(1)TEQGGAHFSVSSLAEGSVTSVGSVNPAENFR | 0.6 | 0.046611 |
| XRCC5 | 338K | 0.00014 | 142.51 | SEGK(1)CFSVLGFCK | 0.596 | 0.000109 |
| XRCC4 | 178K | 0.000729 | 105.36 | EALETDLYK(1)R | 0.975 |  |
| XRCC4 | 197K | 0.000893 | 43.613 | SLHNK(1)LLNAAQER | 0.433 |  |
| XRCC1 | 169K | 0.01706 | 65.933 | VTVTK(1)LGQFR | 1.448 |  |
| XRCC1 | 499K | 0.027932 | 54.436 | VAEQK(1)EHR | 0.783 |  |
| TP53BP1 | 868K | 1.35E-12 | 187.47 | TSNSLTEDSK(1)MANAK | 0.6 | 0.03348 |
| RAD50 | 874K | 0.000248 | 121.71 | SEK(1)LQISTNLQR | 1.424 |  |
| RAD50 | 292K | 0.000618 | 147.19 | DNSELEEK(1)MEK | 1.285 | 0.005586 |
| RAD50 | 132K | 0.000199 | 121.71 | VSLSSK(1)CAEIDR | 1.243 | 0.096153 |
| RAD50 | 599K | 0.001185 | 84.999 | LNK(1)ELASSEQNK | 1.216 | 0.049726 |
| RAD50 | 926K | 0.000498 | 172.1 | FQQEK(1)EELINK | 1.202 | 0.122371 |
| RAD50 | 273K | 0.001532 | 90.689 | LDNEIK(1)ALDSR | 1.185 |  |
| RAD50 | 1032K | 0.000399 | 136.06 | NEELK(1)EVEEER | 1.164 | 0.084252 |
| RAD50 | 969K | 7.02E-06 | 128.41 | DIENYIQDGK(1)DDYK | 1.155 | 0.298275 |
| RAD50 | 1190K | 1.37E-05 | 102.03 | VVMLK(1)GDTALDMR | 1.144 | 0.064394 |
| RAD50 | 267K | 0.022349 | 78.816 | IMK(1)LDNEIK | 1.086 |  |
| RAD50 | 175K | 0.000311 | 90.142 | QK(1)FDEIFSATR | 1.082 | 0.462684 |
| RAD50 | 295K | 4.57E-16 | 99.909 | MEK(1)VFQGTDEQLNDLYHNHQR | 1.02 | 0.717334 |
| RAD50 | 753K | 0.003218 | 134.85 | NK(1)LQNVNR | 1.018 | 0.84245 |
| RAD50 | 845K | 2.21E-08 | 232.17 | LDTVSSK(1)IELNR | 1.015 | 0.851706 |
| RAD50 | 511K | 2.21E-12 | 171.2 | MEVISLQNEK(1)ADLDR | 0.985 | 0.871292 |
| RAD50 | 745K | 0.007391 | 79.059 | EK(1)EIPELR | 0.98 | 0.832451 |
| RAD50 | 421K | 0.000133 | 70.598 | TANQLMNDFAEK(1)ETLK | 0.971 |  |
| RAD50 | 238K | 0.00753 | 90.689 | EAQLTSSK(1)EIVK | 0.942 |  |
| RAD50 | 803K | 0.000142 | 122.51 | FQMELK(1)DVER | 0.912 | 0.521294 |
| RAD50 | 1089K | 0.000474 | 135.63 | GYEEEIIHFK(1)K | 0.897 | 0.111694 |
| RAD50 | 71K | 1.48E-10 | 133.93 | GNTFVHDPK(1)VAQETDVR | 0.896 | 0.037648 |
| RAD50 | 713K | 0.000672 | 114.39 | LK(1)STESELK | 0.894 | 0.166251 |
| RAD50 | 493K | 0.009689 | 77.157 | AEK(1)NSNVETLK | 0.892 |  |
| RAD50 | 446K | 0.000718 | 132.44 | IIELK(1)SEILSK | 0.881 | 0.012736 |
| RAD50 | 832K | 4.46E-05 | 154.94 | TVQQVNQEK(1)QEK | 0.88 | 0.093061 |
| RAD50 | 815K | 1.67E-05 | 115.35 | IAQQAAK(1)LQGIDLDR | 0.868 |  |
| RAD50 | 909K | 5.49E-08 | 167.01 | DAK(1)EQVSPLETTLEK | 0.856 | 0.05319 |
| RAD50 | 461K | 2.23E-12 | 116.36 | NVK(1)YELQQLEGSSDR | 0.831 | 0.491797 |
| RAD50 | 427K | 0.003209 | 121.67 | QK(1)QIDEIR | 0.808 | 0.061341 |
| RAD50 | 743K | 0.0041 | 68.49 | QSIIDLK(1)EK | 0.808 |  |
| RAD50 | 398K | 0.018289 | 84.699 | NFHK(1)LVR | 0.799 | 0.018853 |
| RAD50 | 871K | 0.001965 | 113.46 | STTNELK(1)SEK | 0.795 | 0.013738 |
| RAD50 | 256K | 2.4E-05 | 96.946 | LK(1)EIEHNLSK | 0.779 |  |
| RAD50 | 264K | 0.000335 | 135.28 | EIEHNLSK(1)IMK | 0.753 | 0.000873 |
| RAD50 | 1054K | 4.34E-05 | 106.47 | EMGQMQVLQMK(1)SEHQK | 0.743 | 0.009513 |
| RAD50 | 1139K | 9.43E-05 | 110.88 | FHSMK(1)MEEINK | 0.723 | 0.009378 |
| RAD50 | 976K | 0.000591 | 190.93 | QK(1)ETELNK | 0.712 | 0.051104 |
| RAD50 | 112K | 0.000151 | 104.04 | TEFK(1)TLEGVITR | 0.696 | 0.084921 |
| RAD50 | 583K | 0.000536 | 110.88 | QLEDWLHSK(1)SK | 0.666 | 0.004389 |
| RAD50 | 1059K | 6.58E-05 | 93.162 | SEHQK(1)LEENIDNIK | 0.656 | 0.086308 |
| RAD50 | 343K | 0.000169 | 91.993 | LLNQEK(1)SELLVEQGR | 0.605 | 0.016282 |
| RAD50 | 616K | 0.049935 | 83.358 | NHINNELK(1)R | 0.521 |  |
| PRKDC | 525K | 6.53E-05 | 121.96 | VPTYK(1)DYVDLFR | 2.151 | 0.01539 |
| PRKDC | 1869K | 0.007023 | 95.656 | LNESTFDTQITK(1)K | 1.248 | 0.400844 |
| PRKDC | 801K | 1.29E-15 | 86.952 | DILPCLDGYLK(1)TSALSDETK | 1.244 | 0.50165 |
| PRKDC | 1334K | 0.000532 | 139.78 | YNYSK(1)CTVVVR | 1.24 | 0.328128 |
| PRKDC | 2738K | 0.000408 | 117.99 | DQEK(1)LSLMYAR | 1.165 | 0.506843 |
| PRKDC | 2764K | 3.18E-12 | 237.92 | MK(1)QDAQVVLYR | 1.134 | 0.365685 |
| PRKDC | 2445K | 0.007008 | 54.584 | MMPK(0.989)LK(0.011)PVELR | 1.132 | 0.385998 |
| PRKDC | 117K | 1.58E-05 | 188.6 | NTCTSVYTK(1)DR | 1.115 | 0.553736 |
| PRKDC | 374K | 7.3E-05 | 103.87 | GYGLFAGPCK(1)VINAK | 1.114 | 0.63666 |
| PRKDC | 2369K | 0.008137 | 79.889 | VTK(1)SFPPLADR | 1.1 | 0.892496 |
| PRKDC | 681K | 0.000118 | 96.957 | IK(1)YFEGVSPK | 1.091 | 0.635959 |
| PRKDC | 2683K | 1.31E-44 | 122.21 | SSFDWLTGSSTDPLVDHTSPSSDSLLFAHK(1)R | 1.089 | 0.599006 |
| PRKDC | 689K | 0.000393 | 113.46 | YFEGVSPK(1)SLK | 1.077 | 0.669777 |
| PRKDC | 2132K | 0.000726 | 75.474 | FLHGK(1)LGNPIVPLNIR | 1.06 |  |
| PRKDC | 493K | 0.001319 | 83.358 | ICSK(1)PVVLPK | 1.011 | 0.967043 |
| PRKDC | 4070K | 4.75E-45 | 138.44 | LAGANPAVITCDELLLGHEK(1)APAFR | 1.005 | 0.945221 |
| PRKDC | 163K | 0.000885 | 125.5 | FYGELALK(1)K | 0.996 | 0.949674 |
| PRKDC | 1042K | 0.000395 | 140.01 | WSIK(1)QITPQQQEK | 0.995 |  |
| PRKDC | 1886K | 0.006507 | 69.915 | LPK(1)DDVHAK | 0.99 | 0.970155 |
| PRKDC | 3681K | 5.16E-05 | 64.56 | DSK(0.002)PPGNLK(0.998)ECSPWMSDFK | 0.989 | 0.870934 |
| PRKDC | 263K | 0.000881 | 114.39 | AIRPQIDLK(1)R | 0.986 | 0.786382 |
| PRKDC | 499K | 7.06E-05 | 72.327 | PVVLPK(1)GPESESEDHR | 0.986 | 0.881194 |
| PRKDC | 1209K | 0.000309 | 157.77 | SPNLWLK(1)DVLK | 0.985 | 0.910681 |
| PRKDC | 2715K | 0.001683 | 65.879 | LGLPGDEVDNK(1)VK | 0.982 | 0.801463 |
| PRKDC | 1612K | 0.001062 | 117.39 | ANQK(1)HQGLK | 0.978 | 0.929742 |
| PRKDC | 1857K | 5.82E-54 | 285.55 | FTK(1)LNESTFDTQITK | 0.971 | 0.830213 |
| PRKDC | 148K | 0.00023 | 147.02 | LMDEFK(1)IGELFSK | 0.959 | 0.817271 |
| PRKDC | 1892K | 0.007221 | 65.933 | DDVHAK(1)ESK | 0.957 | 0.784747 |
| PRKDC | 828K | 0.003578 | 108.92 | GFNK(1)VVLK | 0.929 | 0.643404 |
| PRKDC | 4019K | 0.00321 | 125.98 | NFEQK(1)MLK | 0.926 | 0.617246 |
| PRKDC | 329K | 1.02E-07 | 149.58 | QVSNMVAK(1)NAEMHK | 0.921 | 0.592325 |
| PRKDC | 2928K | 0.000293 | 133.98 | WVELAK(1)LYR | 0.92 | 0.606003 |
| PRKDC | 810K | 1.35E-08 | 85.742 | TSALSDETK(1)NNWEVSALSR | 0.914 | 0.672404 |
| PRKDC | 3598K | 0.001512 | 94.804 | AELAK(1)TPVNK | 0.902 | 0.636898 |
| PRKDC | 1412K | 8.47E-05 | 117.21 | MSPYK(1)DILETHLR | 0.894 | 0.510484 |
| PRKDC | 4014K | 0.00041 | 120.65 | EPSFDWK(1)NFEQK | 0.893 | 0.340825 |
| PRKDC | 2824K | 0.006689 | 117.39 | FK(1)TLSEK | 0.889 | 0.511335 |
| PRKDC | 1617K | 2.59E-05 | 137.58 | HQGLK(1)LATTILQHWK | 0.878 | 0.658281 |
| PRKDC | 2447K | 0.006908 | 121.31 | LK(1)PVELR | 0.874 | 0.448494 |
| PRKDC | 2246K | 5.19E-13 | 154.09 | TLVECWK(1)DCLSIPYR | 0.869 | 0.104754 |
| PRKDC | 3264K | 0.012762 | 81.642 | ELHK(1)ESK | 0.867 | 0.467884 |
| PRKDC | 309K | 0.00069 | 113.46 | WCAHTNVELK(1)K | 0.86 | 0.30916 |
| PRKDC | 1627K | 7.97E-13 | 230.73 | LATTILQHWK(1)K | 0.86 | 0.413536 |
| PRKDC | 4085K | 0.004776 | 103.8 | GSK(1)DHNIR | 0.856 | 0.479893 |
| PRKDC | 3260K | 0.012574 | 90.308 | LLK(1)ELHK | 0.855 | 0.402226 |
| PRKDC | 2259K | 0.002876 | 85.716 | LIFEK(1)FSGK | 0.855 | 0.490225 |
| PRKDC | 2970K | 3.17E-15 | 148.57 | SDYSEAAK(1)QYDEALNK | 0.852 | 0.461914 |
| PRKDC | 520K | 0.007003 | 113.14 | WK(1)VPTYK | 0.846 | 0.508799 |
| PRKDC | 3753K | 7.42E-05 | 133.49 | EHPFLVK(1)GGEDLR | 0.843 | 0.385285 |
| PRKDC | 216K | 0.000734 | 78.932 | EPK(1)LPVLAGCLK | 0.821 | 0.367961 |
| PRKDC | 3241K | 0.003616 | 111.39 | MK(1)MIDSAR | 0.798 | 0.264975 |
| PRKDC | 335K | 0.00321 | 106.93 | NAEMHK(1)NK | 0.791 | 0.347029 |
| PRKDC | 1456K | 0.000226 | 119.94 | LAAVVSACK(1)QLHR | 0.773 | 0.255303 |
| PRKDC | 2359K | 7.64E-05 | 84.999 | QHQNTMEDK(1)FIVCLNK | 0.753 | 0.349426 |
| PRKDC | 2221K | 7.82E-05 | 65.933 | LLNFLMK(1)HVFHPK | 0.626 | 0.102019 |
| PCNA | 254K | 5.72E-06 | 183.57 | YYLAPK(1)IEDEEGS | 1.756 | 0.089717 |
| PCNA | 110K | 5.63E-77 | 224.47 | AEDNADTLALVFEAPNQEK(1)VSDYEMK | 1.362 | 0.033525 |
| PCNA | 138K | 8.27E-39 | 122.97 | LMDLDVEQLGIPEQEYSCVVK(1)MPSGEFAR | 1.274 | 0.039289 |
| PCNA | 164K | 5.62E-09 | 141.87 | DLSHIGDAVVISCAK(1)DGVK | 1.085 | 0.336968 |
| PCNA | 13K | 0.001753 | 90.761 | LVQGSILK(1)K | 0.997 |  |
| PCNA | 248K | 6.51E-05 | 70.195 | IADMGHLK(1)YYLAPK | 0.951 | 0.567844 |
| PCNA | 80K | 2.82E-08 | 168.43 | ILK(1)CAGNEDIITLR | 0.921 | 0.150979 |
| PARP1 | 600K | 2.09E-15 | 157.67 | VGTVIGSNK(1)LEQMPSK | 1.396 |  |
| PARP1 | 683K | 0.000245 | 99.855 | MIFDVESMK(1)K | 1.236 | 0.094388 |
| PARP1 | 653K | 1.2E-18 | 200.9 | FYPLEIDYGQDEEAVK(1)K | 1.229 | 0.016525 |
| PARP1 | 447K | 0.00621 | 92.445 | KMEEVK(1)EANIR | 1.226 | 0.306558 |
| PARP1 | 30K | 0.000319 | 90.142 | CSESIPK(1)DSLR | 1.207 | 0.108421 |
| PARP1 | 571K | 1.51E-17 | 182.52 | GTNSYYK(1)LQLLEDDKENR | 1.198 | 0.03147 |
| PARP1 | 262K | 2.18E-13 | 187.47 | VCSTNDLK(1)ELLIFNK | 1.155 | 0.123866 |
| PARP1 | 15K | 0.008229 | 67.507 | VEYAK(1)SGR | 1.086 | 0.128426 |
| PARP1 | 621K | 8.32E-05 | 71.896 | LYEEK(1)TGNAWHSK | 1.044 | 0.623352 |
| PARP1 | 433K | 0.003597 | 98.009 | ASLCISTK(1)K | 1.028 | 0.626501 |
| PARP1 | 518K | 0.005313 | 86.205 | EEGINK(1)SEK | 1.019 |  |
| PARP1 | 108K | 2.98E-08 | 144.2 | AEK(1)TLGDFAAEYAK | 1.01 | 0.881982 |
| PARP1 | 84K | 0.002994 | 129.72 | WDDQQK(1)VK | 1.006 |  |
| PARP1 | 667K | 0.010529 | 57.888 | LPK(1)PVQDLIK | 1.004 | 0.992858 |
| PARP1 | 512K | 1.02E-05 | 195.58 | GQVK(1)EEGINK | 0.983 | 0.790446 |
| PARP1 | 131K | 0.010779 | 93.467 | GCMEK(1)IEK | 0.962 | 0.582721 |
| PARP1 | 796K | 9.41E-34 | 226.73 | GGSDDSSKDPIDVNYEK(1)LK | 0.96 | 0.57558 |
| PARP1 | 949K | 0.006837 | 91.783 | HSVK(1)GLGK | 0.955 | 0.578935 |
| PARP1 | 249K | 4.92E-22 | 253.72 | AQNDLIWNIK(1)DELK | 0.935 | 0.222582 |
| PARP1 | 616K | 4E-05 | 145.08 | EDAIEHFMK(1)LYEEK | 0.922 | 0.07467 |
| PARP1 | 787K | 1.66E-18 | 137.58 | GGSDDSSK(1)DPIDVNYEK | 0.916 | 0.480328 |
| PARP1 | 607K | 9.92E-09 | 113.86 | LEQMPSK(1)EDAIEHFMK | 0.909 | 0.37478 |
| PARP1 | 97K | 7.2E-26 | 151.18 | TAEAGGVTGK(1)GQDGIGSK | 0.902 | 0.323936 |
| PARP1 | 192K | 3.66E-09 | 138 | GFSLLATEDK(1)EALK | 0.875 |  |
| PARP1 | 196K | 0.001055 | 64.266 | GFSLLATEDK(0.038)EALK(0.962)K | 0.86 | 0.141012 |
| PARP1 | 337K | 0.000309 | 133.98 | EWVTPK(1)EFR | 0.844 | 0.056962 |
| PARP1 | 940K | 3.35E-05 | 117.3 | HASHISK(1)LPK | 0.842 | 0.007173 |
| PARP1 | 802K | 7.34E-06 | 95.677 | TDIK(1)VVDRDSEEAEIIR | 0.822 | 0.174909 |
| PARP1 | 105K | 0.00335 | 81.799 | GQDGIGSK(1)AEK | 0.662 | 0.035627 |
| PARP1 | 165K | 0.000664 | 65.156 | WYHPGCFVK(1)NR | 0.58 | 0.008838 |
| MRE11 | 393K | 0.000321 | 63.212 | VANPK(1)DIIHFFR | 0.952 | 0.883336 |
| MRE11 | 510K | 0.000444 | 117.45 | QK(1)NTNEEDDEVR | 0.824 | 0.19072 |
| MRE11 | 384K | 0.010552 | 98.009 | FSQK(1)FVDR | 0.742 | 0.071385 |
| MRE11 | 673K | 1.52E-05 | 118.87 | WSSTSSSK(1)IMSQSQVSK | 0.49 | 0.08943 |
| LIG3 | 581K | 0.005221 | 60.55 | PLPFGTLGVHK(1)K | 0.622 |  |
| LIG3 | 253K | 0.011772 | 74.42 | SLSSSK(1)CDPR | 0.486 |  |
| LIG1 | 376K | 5.11E-10 | 108.41 | AEAAEK(1)GDVGLVAENSR | 0.56 | 0.00509 |
| LIG1 | 298K | 0.000318 | 124.46 | TFEK(1)IEEVSAR | 0.871 | 0.125672 |
| ATM | 573K | 0.006555 | 64.064 | SFSLK(1)ESIMK | 0.627 | 0.05973 |
| ATRIP | 86K | 0.008545 | 54.436 | DVSSDHK(1)VHR | 0.839 |  |
| RPA3 | 39K | 5.79E-05 | 57.042 | IHPTGK(1)MFILSDGEGK | 0.628 |  |
| RPA2 | 231K | 0.00011 | 61.23 | ACPRPEGLNFQDLK(1)NQLK | 0.757 |  |
| RPA2 | 93K | 9.88E-17 | 149.57 | APTNIVYK(1)IDDMTAAPMDVR | 0.675 | 0.278351 |
| RPA2 | 243K | 8.86E-14 | 85.764 | HMSVSSIK(1)QAVDFLSNEGHIYSTVDDDHFK | 1.022 |  |
| RPA1 | 595K | 0.000222 | 115.04 | ATVMDVK(1)PVDYR | 1.218 | 0.225628 |
| RPA1 | 163K | 0.027502 | 61.48 | AYGASK(1)TFGK | 0.959 | 0.642135 |
| RPA1 | 88K | 0.000355 | 97.277 | FIVNTLK(1)DGR | 0.808 | 0.157797 |
| RPA1 | 588K | 8.45E-05 | 151.52 | IK(1)ATVMDVK | 0.96 | 0.370025 |
| RPA1 | 577K | 0.000311 | 108.4 | VK(1)VETYNDESR | 1.088 | 0.317816 |

**Table S2 Comparison of differences regarding tumor doubling time among mice treated with or without Ku80 K568R prior to radiation.**

|  | Mean Diff. | P value | Summary |
| --- | --- | --- | --- |
| Ku80 WT vs. Ku80 K568R | -2.1 | 0.0017 | ** |
| Ku80 WT vs. Ku80 IR | -8.0 | < 0.0001. | **** |
| Ku80 WT vs. Ku80 K568R IR | -13.6 | < 0.0001. | **** |
| Ku80 K568R vs. Ku80 WT IR | -5.9 | < 0.0001. | **** |
| Ku80 568Rvs. Ku80 K568R IR | -11.5 | < 0.0001. | **** |
| Ku80 WT IR vs. Ku80 K568R IR | -5.6 | < 0.0001. | **** |

One-way ANOVA and Tukey’s multiple comparisons testing.

** = *p* < 0.01, **** = *p* < 0.0001.

**Original and uncropped films of Western blots**

**Figure 2**

**Figure 3**

**Figure 4**

**Figure 6**

**Figure S2**

**Figure S3**

**Figure S4**

**Figure S5**

**Figure S6**

**Figure S7**

**Figure S8**

**Figure S19**

**Figure S20**

**Figure S22**
